# Danggui Buxue Decoction Alleviates Inflammation and Oxidative Stress in Mice with *Escherichia coli*-Induced Mastitis

**DOI:** 10.3390/vetsci12030227

**Published:** 2025-03-02

**Authors:** Jiamian Wang, Chen Cheng, Yujin Gao, Yina Li, Xijun Zhang, Dan Yao, Yong Zhang

**Affiliations:** 1 College of Veterinary Medicine, Gansu Agricultural University, Lanzhou 730070, China; 17899319313@163.com (J.W.); cheng970318@163.com (C.C.); gyj1234561202@163.com (Y.G.); lyn9097@163.com (Y.L.); junerzhang0325@gmail.com (X.Z.); 15776502994@163.com (D.Y.); 2 Gansu Key Laboratory of Animal Generational Physiology and Reproductive Regulation, Lanzhou 730070, China

**Keywords:** Danggui buxue decoction (DBD), *Escherichia coli* (*E. coli*) mastitis, inflammation, oxidative stress (OS)

## Abstract

(1) Background: Bovine mastitis is a lactational disease caused by infection and milk stagnation in the mammary glands. Danggui buxue decoction (DBD), a traditional remedy for blood tonification, anti-inflammation, and antioxidation, has not been used previously to treat mastitis. (2) Methods: In this study, an *Escherichia coli* mastitis model was established by infecting lactating Kunming mice with clinically isolated bovine mastitis-derived *E. coli*. Based on this, the effects of DBD on inflammation and oxidative stress in mastitis model mice were evaluated by conducting routine blood tests, H&E staining, qRT-PCR analysis, ELISA, and microcolorimetry. (3) Results: We found that DBD treatment reduced body weight loss, abnormal organ indices, abnormal blood cell counts, pathological damage to breast tissue, and the upregulation of the expression of inflammatory factor in mice caused by *E. coli* infection. We also found that DBD increased the expression of antioxidants and antioxidant genes and decreased the expression of oxidation products and oxidation-related genes in breast tissue. The therapeutic effect of DBD on inflammation and oxidative stress (OS) in mice occurred through the regulation of the TLR4/NF-κB and Nrf2/HO-1 signaling pathways. (4) Conclusions: DBD imparted its anti-inflammatory and antioxidant effects by inhibiting the TLR4/NF-κB signaling pathway and activating the antioxidant Nrf2/HO-1 signaling pathway

## Simple Summary

Bovine mastitis (BM) is a common disease in dairy farming, which occurs mostly in the postpartum lactation period of dairy cows. Danggui buxue decoction (DBD), as a traditional prescription for tonifying blood, activating blood, anti-inflammation, and anti-oxidation, has not been applied previously to the treatment of mastitis. In this study, lactating mice were infected with clinically isolated bovine mastitis-derived *Escherichia coli* (*E. coli*), and the *E. coli* mastitis model was successfully established. On this basis, the therapeutic effect of DBD on mastitis mice was evaluated. We found that DBD can effectively control inflammation and oxidative stress in *E. coli*-induced mastitis mice by regulating the TLR4/NF-κB and Nrf2/HO-1 signaling pathways, suggesting that DBD may be a very effective new method for the treatment of bovine mastitis.

## 1. Introduction

Bovine mastitis (BM) is an inflammatory disease caused mainly by the bacterial invasion of mammary ducts during postpartum lactation [1]. On the basis of their clinical symptoms, BM can be categorized into two types: clinical mastitis and subclinical mastitis. *Escherichia coli* (*E. coli*) is a primary bacteria that causes clinical mastitis in bovines. BM induced by *E. coli* is associated with increased transmission and more severe acute reactions [2,3]. The most common way to treat BM is to kill pathogenic bacteria and reduce the inflammatory response. Although commonly used antibiotic therapy can kill pathogenic bacteria and effectively alleviate BM, this treatment has prominent side effects [4]. Therefore, new alternative therapeutic strategies are urgently required, and traditional Chinese herbal medicine has received increasing attention from researchers as an alternative to antibiotics.

As Chinese herbal medicine has a low cost, high efficiency, low toxicity, no drug resistance, is rich in effective substances and many targets, and can improve the immunity and disease resistance of the body, Chinese herbal medicine has become an ideal antibiotic substitute for treating BM [5]. Therefore, traditional Chinese medicine compound preparations for mastitis need to be urgently developed for the sustainable and healthy development of dairy farming. Danggui buxue decoction (DBD) was one of the first classical prescriptions in ancient China. In the prescription, only *Angelica sinensis* and *Astragalus membranaceus* were combined at a 5:1 ratio. Although the prescription is simplified, it has strong efficacy [6]. DBD is widely used in clinical practice and is often used to treat anemia, diabetes, tumors, and cancer [7,8,9]. However, the use of DBD for treating BM is scarce; in order to make up for this research gap, we used DBD to treat mastitis mice to study the therapeutic effect and related mechanisms of DBD. The results can provide data for the study of DBD for the treatment of BM and lay a foundation for the application and promotion of DBD in veterinary clinics.

The mammary glands of cows suffering from clinical mastitis exhibit different degrees of inflammatory changes, such as congestion, redness, atrophy, fever, and tenderness. In severe cases, the mammary glands may suffer permanent damage, and even more severe systemic symptoms may occur [10]. H&E staining can reveal pathological changes, such as a reduction in the acinar cavity, necrosis and shedding of acinar epithelial cells, and an increase in the infiltration of inflammatory cells in the mammary glands of diseased animals [11]. Moreover, the expression of TNF-α, IL-1β, IL-6 mRNAs, and proteins is elevated in the breast tissue of diseased cows [12]. Many researchers have shown that oxidative stress (OS) injury is one of the key ways to promote the occurrence and development of BM [13]. DBD can improve histopathological changes, reduce the expression of inflammatory factors, and alleviate OS. For example, Li et al. reported that the use of DBD to treat inflammatory bowel disease can improve the pathological state of the intestine in diseased mice, promote the regression of inflammation, and significantly promote the proliferation of intestinal epithelial cells [14]. Wang et al. investigated the effect and mode of action of DBD in pulmonary fibrosis (PF) rats and reported that DBD can suppress TNF-α, IL-1β, and IL-6 release and prevent lung fibrosis in these rats [15]. To study the mechanism by which DBD relieves bone marrow suppression (MAC) after chemotherapy, Gao et al. reported that DBD can achieve therapeutic effects by regulating β-hydroxybutyric acid metabolism and inhibiting OS [6]. Li et al. used DBD to treat vascular aging in mice suffering from chronic intermittent hypoxia and reported that DBD treatment inhibited the expression of IL-6, NF-κB, and TNF-α; decreased the content of malondialdehyde (MDA); and increased the activity of superoxide dismutase (SOD). Moreover, this effect on inflammatory processes and OS may be achieved by modulating the Nrf2/HO-1 pathway [7]. Several studies have shown the anti-inflammatory, antioxidation, and immunity-enhancing properties of DBD [16,17]; thus, we hypothesized that DBD might mitigate inflammation and OS in *E. coli*-infected mastitis model mice.

Therefore, in this study, mice infected with clinically isolated BM-derived *E. coli* were used to evaluate whether or not the mastitis mouse model was constructed by anatomical observation, routine blood analysis, H&E staining, and the expression of cytokine proteins and mRNAs. Subsequently, the mice with mastitis were treated with DBD, and the related indicators of inflammation and OS in each group were detected. The expression of the TLR4/NF-κB p65 inflammatory signaling pathway and the Nrf2/HO-1 endogenous antioxidant signaling pathway during treatment was investigated. The results of this study can be used to evaluate the therapeutic effect of DBD on BM and elucidate its underlying mechanism of action, determine new strategies for treating BM, and lay the foundation for using and promoting DBD in veterinary clinics.

## 2. Materials and Methods

### 2.1. Reagents

The sequencing and synthesis of primers were performed by Tsjngke (Beijing, China). Kunming mice (*n* = 50, four weeks old) were purchased from the Lanzhou Veterinary Research Institute (Lanzhou, China). The herbs *Angelica sinensis* and *Astragalus membranaceus* were purchased from Lanzhou, China. The quality of the medicinal materials was assessed under the guidance of the teacher of the Chinese veterinary team. Mouse TNF-α (YX-20220 M), IL-1β (YX-E20533), IL-6 (YX-20012 M), and IL-8 (YX-20459 M) ELISA kits were purchased from Sino Best (Shanghai, China). Luria-Bertani (LB) liquid medium (L1010) and a T-AOC assay kit (BC1315) were obtained from Solarbio (Beijing, China). The GSH assay kit (A006-2-1), SOD assay kit (A001-3), and MDA assay kit (A003-1) were purchased from Nanjing Jiancheng (Nanjing, China). A NO assay kit (S0021S) was obtained from Beyotime (Shanghai, China). The TIANamp Bacteria DNA Kit was purchased from TIANGEN (Beijing, China). The anti-TLR4 antibody (CQA3707) (1:1000) was purchased from Cohesion (London, UK). A rabbit anti-MyD88 antibody (bs-1047R) (1:3000), anti-IKKβ antibody (bs-4880R) (1:6000), and anti-NF-κB p65 antibody (bs-0465R) (1:1000) were purchased from Bioss (Beijing, China). P-NF-κB p65 (Ser536) antibody (TA2006S) (1:2000), P-IκBα (Ser32/Ser36) antibody (TP56280) (1:2000), IκBα antibody (T55026) (1:2000), Nrf2 antibody (T55136F) (1:1000), heme oxygenase 1 antibody (T55113F) (1:1000), and NQO1 antibody (T56710F) (1:2000) were purchased from Abmart Bio (Shanghai, China).

### 2.2. Strains

The *E. coli* used in the experiment was isolated from the milk samples of cows with clinical mastitis and was strictly preserved. Before the experiment, *E. coli* was streaked on LB plate medium, and a single typical colony was transferred to LB liquid medium for expansion culture. The strain was examined via Gram staining microscopy, and the bacterial DNA was extracted via a bacterial genomic DNA extraction kit. PCR amplification was performed via 16S universal primers. The reaction mixture contained 12.5 µL of 2×M5 HiPer plus Taq HIFI PCR mix (with blue dye), 1 µL of each forward and reverse primer, 2 µL of the DNA template, and 8.5 µL of H_2_O. The reaction conditions were 95 °C for 300 s, 35 cycles of 95 °C for 50 s, 55 °C for 50 s, and 72 °C for 60 s. The reaction products were sequenced and submitted to NCBI for comparison. The accession number (KJ803889.1) of the strain was obtained after comparison. The pathogen was used strictly following the “Pathogen Microbiological Laboratory Biosafety Management Regulations”.

### 2.3. Animals

Kunming mice were raised in a specific pathogen-free (SPF) environment at 26 °C with 12 h of light per day, with free access to drinking water and food; all the mice were fed special maintenance feed without antibiotics. Animal experiments were performed after one week of adaptive culture. The sexually mature mice were injected with pregnant mare serum gonadotropin (PMSG) for simultaneous estrus treatment, and the males and females were caged for 3–4 days. Female mice lactating within 3–7 days after delivery and weighing 48 ± 4.5 g were used to establish a mouse mastitis model [18].

### 2.4. Preparation of DBD

**Figure 1:**
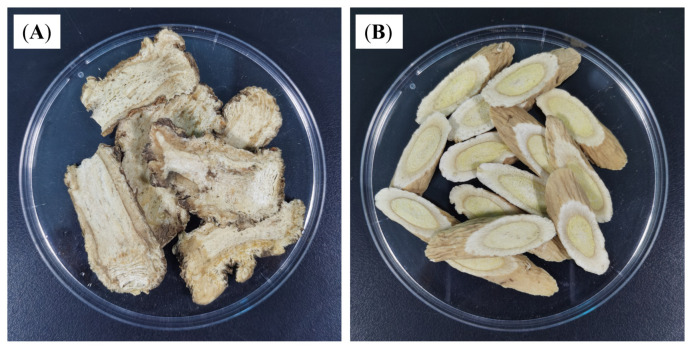
Herbs used to make the DBD are shown. (**A**) *Angelica sinensis*; (**B**) *Astragalus membranaceus*.

The two Chinese herbal medicines that constitute the DBD are ASR and AR, as shown in Figure 1. The DBD concentrate was prepared following a previously described method [19,20]. The concentrated liquid can be stored at 4 °C for one week. The adult dosage of DBD was 36 g·kg^−1^·d^−1^, and the clinical equivalent dose for the mice was 4.5 g·kg^−1^·d^−1^ according to the calculation of the ‘equivalent dose coefficient conversion algorithm of human and animal body surface areas’.

### 2.5. Establishment of the Mastitis Model

**Figure 2:**
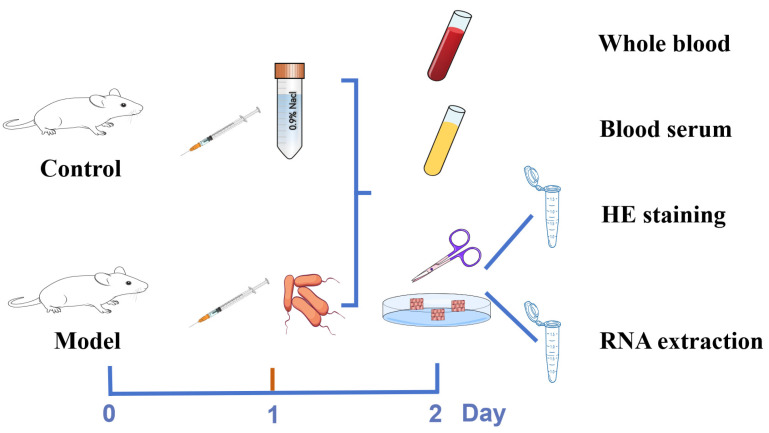
Establishment of a mouse mastitis model induced by *E. coli* and a sample acquisition scheme.

To establish an *E. coli*-induced mouse mastitis model, *E. coli* was used to infect 10 healthy lactating mice. The method used to establish the mouse mastitis model was slightly modified from previous studies [21]. The *E. coli* mixture from dairy cow mastitis sources cultured overnight was collected and counted following the method recommended by the United States CLSI (formerly NCCLS), and the *E. coli* bacterial mixture (5 × 10^3^ CFU/mL) was obtained. Next, 2 h after the pups were removed, 50 μL of bacterial mixture was injected into the left and right sides of the fourth pair of mammary glands of lactating female mice, and the controls were injected with an identical volume of saline. Twenty-four hours after injection, the mental status of the mice was observed, their body weights were measured, and blood samples were collected. Next, the mice were sacrificed and their breast tissues were collected. The experimental protocols for establishing the mouse mastitis model and collecting samples are shown in Figure 2. The blood parameters were evaluated via routine blood tests, pathological changes in the breast tissues were visualized via H&E staining, and the expression of inflammatory factors in the serum or tissues was detected via ELISA and qRT-PCR. These indicators were used to evaluate whether or not the mastitis mouse model was successfully established.

### 2.6. Grouping and Sample Collection for DBD Treatment of Mastitis in Mice

**Figure 3:**
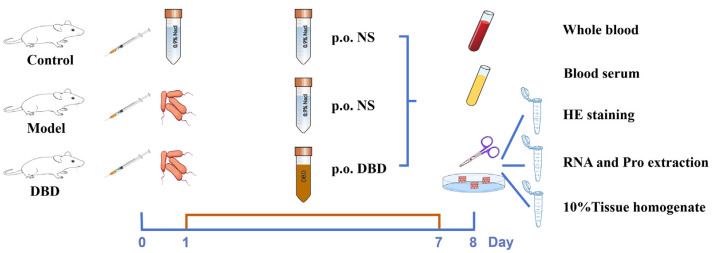
Experimental protocol for DBD treatment of mastitis in mice and sample collection.

The lactating mice (*n* = 30) were divided into three groups (*n* = 10): the control group (containing 100 μL of normal saline and gavaged with 1 mL of normal saline·d^−1^), the model group (containing 100 μL of bacterial mixture (5 × 10^3^ CFU/mL) and gavaged with 1 mL of normal saline·d^−1^), and the DBD treatment group (containing 100 μL of bacterial mixture (5 × 10^3^ CFU/mL) and gavaged with 9 mg·g^−1^·d^−1^ DBD). The experimental protocol for the DBD treatment of mastitis in mice and sample collection is shown in Figure 3. The blood of each mouse was collected in a heparin sodium anticoagulant tube and a centrifuge tube, and the hematological parameters were measured after the blood in the anticoagulant tube was mixed evenly. The blood in the centrifuge tube was incubated for 30 min and centrifuged at 4 °C and 3000 rpm/min for 10 min to obtain serum for peripheral blood cytokine detection. The breast tissue was collected, the blood and latex were washed with phosphate-buffered saline (PBS) after the fat was removed, and the breast tissue was divided into three portions. One sample was used for H&E staining, one was used to extract RNA for qRT-PCR analysis, and one was added to normal saline to make a 10% tissue homogenate.

### 2.7. Body Weight and Organ Indices

The body weight, thymus weight, spleen weight, and liver weight of the mice were recorded. The percentage weight reduction and organ index were calculated [22]. Percentage weight reduction = (starting weight − end weight)/starting weight − 100%; Organ index = Organ index weight (mg)/body weight (g) × 100%.

### 2.8. Blood Routine

The blood in the anticoagulant tube was mixed evenly with the anticoagulant, and the hematological parameters were measured via an automatic blood cell analyzer (Mindray Animal Medical, Shenzhen, China).

### 2.9. H&E Staining

Following conventional methods, a fourth pair of mouse breast tissue was collected for histopathological observation. The breast tissue was fixed, dehydrated, waxed, embedded, sliced, dewaxed, stained, sealed, and observed under a microscope.

### 2.10. ELISA

The 10% tissue homogenate was centrifuged at 3500 rpm/min for 15 min. The homogenate supernatant and serum were cryopreserved, and the cytokine content in each sample was detected via an ELISA kit within one week. Following the instructions of the ELISA kit, 50 μL of sample mixture was added to the blank well (2 replicates), 50 μL of a standard mixture of different concentrations was added to the standard well (2 replicates) to generate the standard curve, and 10 μL of sample mixture and 40 μL of sample mixture were added to the sample well (1 replicate for each serum sample and 2 replicates for each tissue sample). Except for the blank well without the antibody, the other wells were incubated with the antibody, and the reactions were colored and terminated. Finally, the OD value of each well at the corresponding wavelength was detected and the content of cytokines in the sample was calculated.

### 2.11. qRT-PCR and Western Blot (WB) Assays

RNA was extracted from the breast tissue, and the cDNA obtained via reverse transcription was used for qRT-PCR analysis. The extracted protein was used for WB analysis. The specific steps were similar to those of previous studies; only the primers and antibodies used were different [20]. The primers used are listed in Table 1. The brief steps of WB include collecting samples and performing lysis and denaturation. The denatured product was subjected to SDS-PAGE, and then the protein on the gel was subsequently transferred to a PVDF membrane via the wet transfer method, after which the PVDF membrane was placed in the blocking solution for blocking. After blocking, the membrane was incubated with the appropriate antibody, and the band was subsequently exposed via an Amersham ImageQuant 800 (Cytiva, Tokyo, Japan). Finally, gray analysis was performed via ImageJ 1.48v software (National Institutes of Health, Bethesda, MD, USA). Raw Western blot data with molecular weight markers are presented in Supplementary S1.

### 2.12. Oxidation and Antioxidant Indices

The total antioxidant capacity (T-AOC), glutathione (GSH) content, superoxide dismutase (SOD) activity, malondialdehyde (MDA) content, and nitric oxide (NO) content of each sample were determined according to the instructions of the kit.

### 2.13. Statistical Analysis

GraphPad Prism 8.4.2 was used to analyze the data statistically. The results are expressed as the mean ± SEM. The mouse mastitis model was evaluated via t-tests; DBD treatment was assessed via one-way ANOVA. Tukey’s honestly significant difference test (Tukey HSD) was used to adjust the results of multiple comparisons. *p* < 0.05 was considered to indicate statistical significance; *p* < 0.01 indicated high statistical significance. “*” indicates *p* < 0.05 compared with the control group, and “**” indicates *p* < 0.01 compared with the control group. “#” indicates *p* < 0.05 compared with the model group and “##” indicates *p* < 0.01 compared with the model group.

## 3. Results

### 3.1. Establishing the Mastitis Model

**Figure 4:**
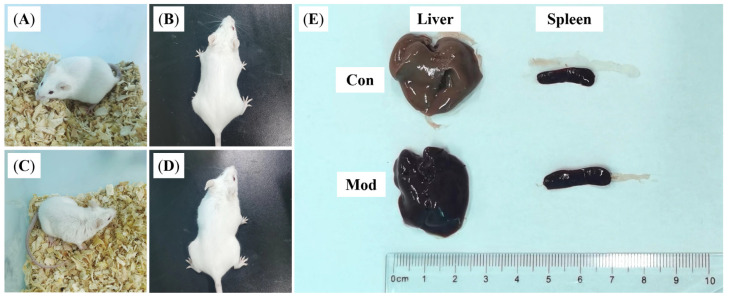
Appearance and internal organs of the mice. (**A**,**B**) Control group; (**C**,**D**) Model group; (**E**) Liver and spleen of the mice.

**Figure 5:**
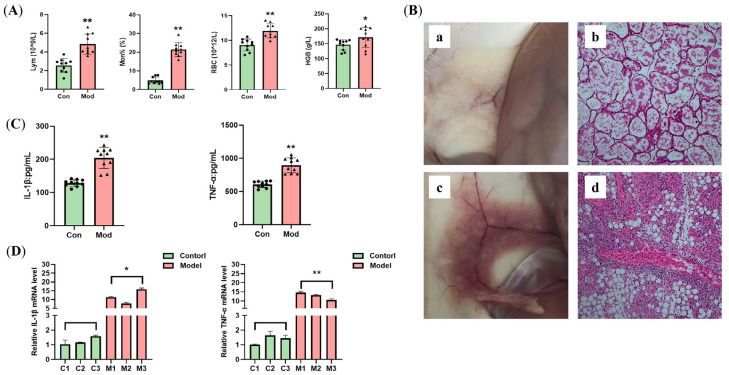
*Escherichia coli*-induced mastitis in mice. (**A**) Hematological parameters of the mice; (**B**) H&E staining (100×), (**a**,**b**) Control group, (**c**,**d**) Model group; (**C**) Cytokine levels in the serum; (**D**) Relative mRNA expression in the mouse mammary tissue. “*” indicates *p* < 0.05 compared with the control group, and “**” indicates *p* < 0.01 compared with the control group.

The control group mice are shown in Figure 4A,B. The mice in the control group had a good mental state, rapid response, a clean and tidy coat, and a normal posture. The model group is shown in Figure 4C,D. One day after the injection of *E. coli*, the mice were depressed, their eyes were lax, their reactions were slow, their coats were messy, and their abdomens were depressed. After the mice were dissected, the livers of the mice in the model group appeared to be congested, blood stasis occurred, the color of the liver was dark red in ocular view, and the spleen was abnormally enlarged (Figure 4E). The results of the peripheral blood parameters are shown in Figure 5A. The Lym and Mon% in the blood of the mice infected with *E. coli* were significantly greater than those in the reference group (*p* < 0.01), and the numbers of red blood cells (RBCs) and hemoglobin (HGB) were significantly greater (*p* < 0.05), suggesting that inflammation, a reduction in the plasma volume, and hypoxia occurred in these mice. To visually assess the pathogenic alterations in the breast tissue, H&E staining was performed. As shown in Figure 5B, the mammary gland tissue (Figure 5B(a,b)) of the control group was milky white. H&E staining revealed that the healthy mouse mammary gland follicles were structurally intact, with abundant glandular fluid in the lumen of the follicles. The mammary tissue of mice infected with *E. coli* was swollen, red, and congested. As shown in Figure 5B(c,d), the staining results also revealed that the wall of the mammary gland acinar was disrupted, the integrity of the acinar was destroyed, and there was massive inflammatory cell infiltration in the tissue, vascular congestion, and the presence of several inflamed cells in the blood vessels. The cytokine levels in the serum are shown in Figure 5C, and the relative mRNA expression in the breast tissue is shown in Figure 5D. These results indicated that *E. coli* infection of mouse breast tissue increased the number of inflammatory cells in mouse peripheral blood and breast tissue. These results indicated that the construction of the *E. coli*-induced mastitis mouse model was successful and that this model can be used in subsequent experiments.

### 3.2. Effects of DBD on the Body Weight and Organ Indices of Mastitis Model Mice

**Figure 6:**
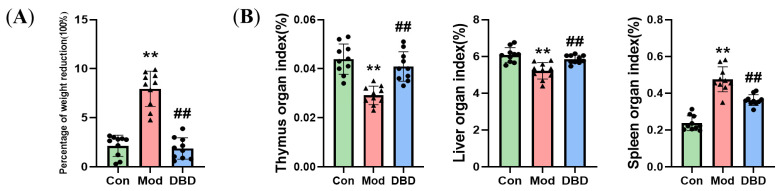
Weight and Organ index. (**A**) Percentage weight reduction = (starting weight − end weight)/starting weight − 100%; (**B**) Organ index = Organ index weight (mg)/body weight (g) × 100%. “**” indicates *p* < 0.01 compared with the control group. “##” indicates *p* < 0.01 compared with the model group.

After seven days of DBD administration, the mice’s body weights in the three treatment groups decreased. The weight loss percentage of the model group was considerably greater than that of the control group. Compared with that in the infected group, the percentage of weight loss in the mice that were administered DBD was significantly lower, indicating that DBD can alleviate the weight loss caused by *E. coli* infection (Figure 6A). The livers and spleens of the mice in each group were weighed, and the organ indices were computed (Figure 6B). The findings revealed that the mice in the mastitis group had a decreased thymus coefficient and liver coefficient (*p* < 0.01) compared with those in the normal group. Additionally, the spleen coefficient (*p* < 0.01) was greater than that of the mice in the normal group, whereas the organ coefficient of the mice in the DBD group was normal; these findings indicate that DBD can alleviate the abnormal functions caused by *E. coli* infection in the organs of these mice.

### 3.3. Effects of DBD on Hematological Parameters in Mastitis Model Mice

**Figure 7:**
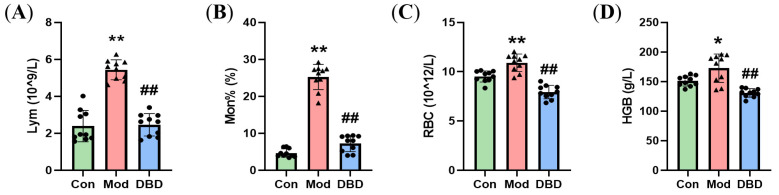
Hematological parameters of the mice. (**A**) Lym; (**B**) Mon%; (**C**) RBCs; (**D**) HGB. “*” indicates *p* < 0.05 compared with the control group, and “**” indicates *p* < 0.01 compared with the control group. “##” indicates *p* < 0.01 compared with the model group.

The routine blood test results of the DBD-treated mice with mastitis are shown in Figure 7. *Escherichia coli* infection significantly increased the numbers of lymphocytes, monocytes, erythrocytes, and hemoglobin in the blood of mice with mastitis (*p* < 0.05). Compared with those in the *E. coli*-treated group, these indices were significantly lower in the group of mice gavaged with DBD (*p* < 0.01).

### 3.4. DBD Alleviates Histopathological Changes in Mastitis Model Mice

**Figure 8:**
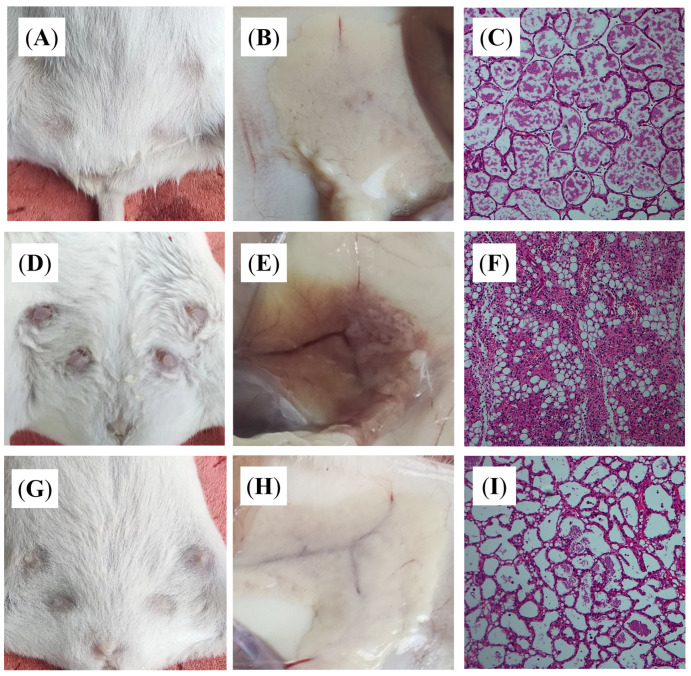
Eye view and H&E staining (100×). (**A**–**C**) Control group; (**D**–**F**) Mastitis group; (**G**–**I**) DBD group.

We assessed the effect of DBD on the mammary tissue of mastitis model mice by observing it with the naked eye and examining H&E-stained sections. In mastitis-free mice (Figure 8A–C), the breast tissue structure was intact, the acinar boundary was distinct, and no pathological changes were detected. After the mice were infected with *E. coli* (Figure 8D–F), the breasts of the mice were hard, the nipples were red and swollen, the breast tissue was congested, and the acinar structure was destroyed due to the infiltration of inflammatory cells. In the DBD group (Figure 8G–I), the number of inflammatory cells decreased, the severity of tissue injury was alleviated, and the degree of pathological alterations also decreased.

### 3.5. DBD Attenuates the Inflammatory Response in Mastitis Model Mice

**Figure 9:**
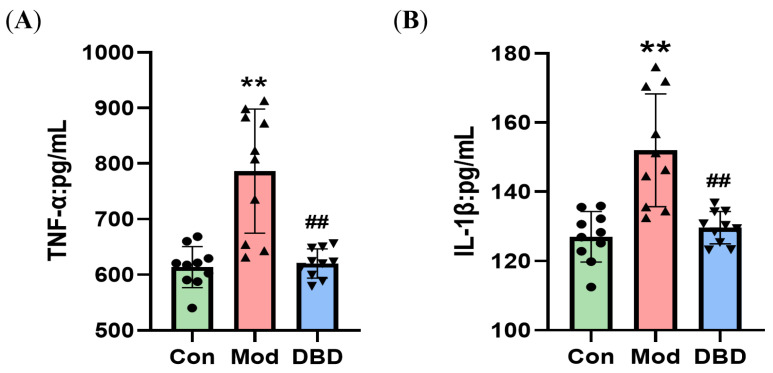
ELISA was performed to determine the levels of TNF-α and IL-1β in the serum. (**A**) TNF-α; (**B**) IL-1β. “**” indicates *p* < 0.01 compared with the control group. “##” indicates *p* < 0.01 compared with the model group.

**Figure 10:**
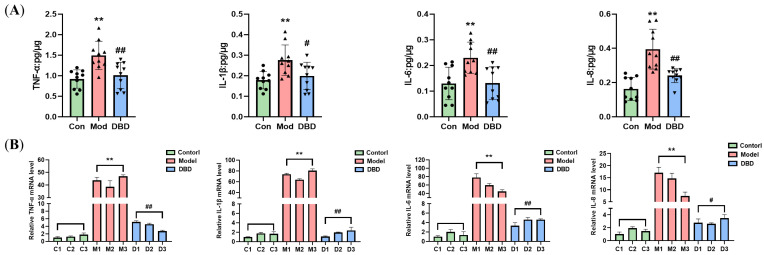
Levels of inflammatory factors in mammary tissues. (**A**) ELISA; (**B**) qRT-PCR. “**” indicates *p* < 0.01 compared with the control group. “#” indicates *p* < 0.05 compared with the model group and “##” indicates *p* < 0.01 compared with the model group.

To determine the effect of DBD on inflammation in mastitis mice, we used an ELISA kit to measure the levels of cytokines in the serum and breast tissue of the mice and performed qPCR analysis to test the mRNA levels of inflammatory factors in the tissues. We found that the concentrations of TNF-α and IL-1β in the serum of mice were lower (*p* < 0.01) (Figure 9), and the protein and mRNA levels of inflammatory cells in the breast tissue decreased to different degrees (*p* < 0.05 or *p* < 0.01) after treatment with DBD (Figure 10).

### 3.6. Effects of DBD on the TLR4/NF-κB Signaling Pathway

**Figure 11:**
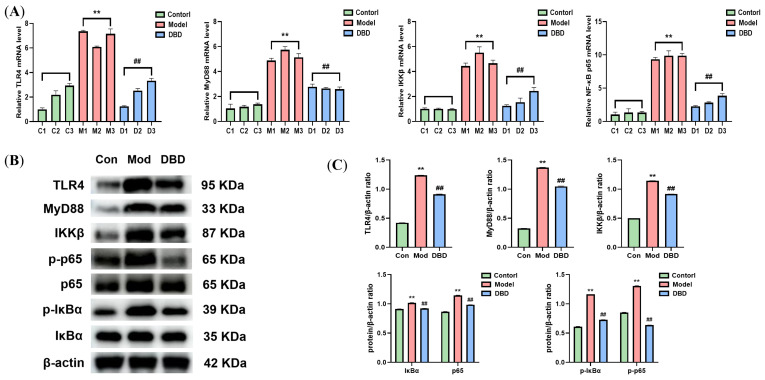
The effect of DBD on the TLR4/NF-κB signaling pathway. (**A**) mRNA levels; (**B**) protein levels; (**C**) gray analysis. “**” indicates *p* < 0.01 compared with the control group. “##” indicates *p* < 0.01 compared with the model group.

As shown in Figure 11, WB and qRT-PCR analyses of the key factors involved in the TLR4/NF-κB inflammatory signaling pathway were conducted. The mRNA levels of TLR4, MyD88, IKKβ, and NF-κB p65 were significantly different between the model group and the non-mastitis group (*p* < 0.01). Moreover, the expression levels of the TLR4, MyD88, and IKKβ proteins in the pathway increased significantly, and the phosphorylation of the NF-κB p65 and IκBα proteins also increased significantly (*p* < 0.01). After DBD therapy, the expression and activation of the TLR4/NF-κB signaling pathway are inhibited.

### 3.7. DBD Alleviates OS in Mastitis Model Mice

**Figure 12:**
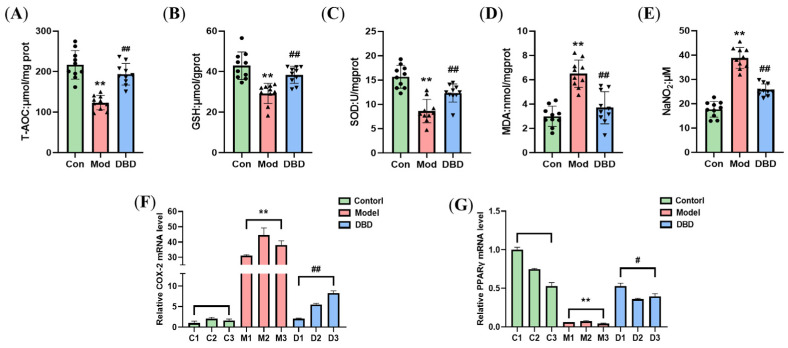
Oxidative and antioxidant indices in mouse mammary tissues. (**A**) T-AOC of mammary tissues; (**B**) GSH content of mammary tissues; (**C**) SOD activity of mammary tissues; (**D**) MDA content of mammary tissues; (**E**) NO level in serum; (**F**,**G**) relative mRNA levels of COX-2 and PPARγ. “**” indicates *p* < 0.01 compared with the control group. “#” indicates *p* < 0.05 compared with the model group and “##” indicates *p* < 0.01 compared with the model group.

To assess the effects of DBD on the OS of mastitis model mice, we determined the oxidative and antioxidant indices in the tissues and serum (Figure 12). DBD increased the total antioxidant capacity, glutathione content, and superoxide dismutase activity of mammary tissue in mastitis model mice and decreased malondialdehyde production in mammary tissue and nitric oxide levels in the serum (*p* < 0.01). Moreover, DBD decreased the expression of COX-2 mRNA (*p* < 0.01) and increased the expression of PPARγ mRNA (*p* < 0.05).

### 3.8. Effects of DBD on the Nrf2/HO-1 Signaling Pathway

**Figure 13:**
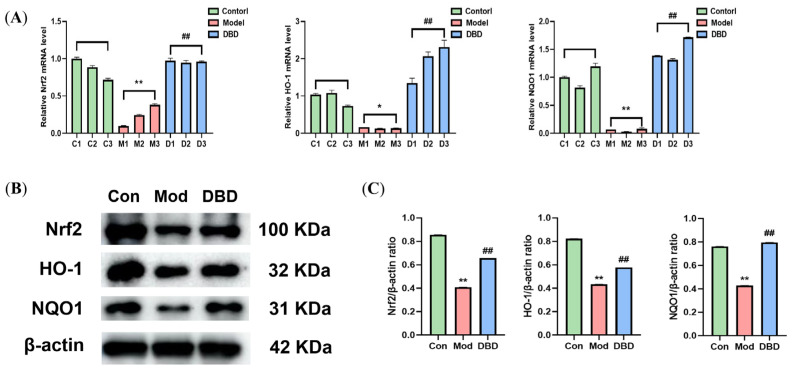
The effect of DBD on the Nrf2/HO-1 signaling pathway. (**A**) mRNA levels; (**B**) protein levels; (**C**) gray analysis. “*” indicates *p* < 0.05 compared with the control group, and “**” indicates *p* < 0.01 compared with the control group. “##” indicates *p* < 0.01 compared with the model group.

Inflammation caused by *E. coli* stimulation is strongly associated with OS. To investigate the effect of DBD treatment on the activation of the endogenous antioxidant stress signaling pathway Nrf2/HO-1, we performed qPCR and WB analyses to detect the mRNA and protein expression of Nrf2, HO-1, and NQO1, as shown in Figure 13. The expression of Nrf2, HO-1, and NQO1 was detected in the control group. The expression of antioxidant genes and proteins decreased considerably after *E. coli* stimulation of mammary glands (*p* < 0.05), whereas DBD treatment increased their expression.

## 4. Discussion

Bovine mastitis is mostly local inflammation caused by pathogenic bacteria that invade mammary ducts. The disease has a high incidence and causes considerable economic losses, which severely restricts the development of the dairy cattle breeding industry [23]. Antibiotic therapy, which has several side effects, such as bacterial resistance and antibiotic residues [24], is mostly used for treating mastitis. To avoid these risks, veterinary researchers and practitioners have investigated new alternative therapeutic techniques. DBD is one of the first batches of ‘ancient classic prescription catalogs’ published in China and is a commonly used remedy for tonifying qi and promoting blood circulation [25]. DBD is widely used in clinical practice. It can promote hematopoiesis, regulate immunity, fight inflammation, and regulate OS. However, research on the application of DBD for treating BM is lacking. Owing to their high cost, long cycle, and difficulty in clinical research, animal models are commonly used to study the pathogenesis of diseases. Therefore, by establishing a mouse mastitis model, the research cost can be reduced, the research efficiency can be improved, and research progress can be accelerated [26]. Therefore, in this study, *E. coli* was used to infect lactating mice to establish an animal model of mastitis, and the protective effect of DBD on mastitis in mice was assessed in this model, which provides data for the application of DBD in veterinary clinics.

Body weight is an overall indicator that reflects the health status and nutritional status of the body, and the organ index is an important indicator reflects the size, functional status, and development of organs. The mice infected with *E. coli* were unwell and experienced weight loss. Moreover, the livers of the mice in the mastitis group were congested, and the liver index was reduced, the spleen was abnormally enlarged, and the thymus index decreased. When DBD was administered orally to mice with mastitis, weight loss and abnormal organ indices were alleviated. This result was also reflected in the studies by Li et al., who used DBD to treat cyclophosphamide-induced immunosuppression in broiler chickens, and Liu, who experimentally treated immunocompromised colon injury in rats with *Angelica sinensis* polysaccharides [27,28]. The process of lactation relies on blood circulation, and the liver, as the largest blood-producing organ, once damaged, leads to qi and blood deficiency and affects milk secretion [29]. Moreover, the obstruction of the hepatic portal vein can cause splenomegaly. The spleen, as the main immune organ, produces immunoglobulins, complement, and lymphocytes, which impart immunity; thus, abnormal spleen function reduces the immunity of the body [30]. The thymus coefficient can reflect lymphocyte proliferation and immunity. A thymus coefficient that is too low indicates thymic dysfunction and low immunity. Overall, DBD can ameliorate weight loss and organ damage in diseased mice, promote blood circulation in the body, and improve immunity.

In addition to body weight and organ indices, a number of indicators in routine blood tests can also reflect pathological changes within the body. Therefore, we used peripheral blood variables to evaluate the physiological characteristics of the mice. The percentages of Lym and Mon% in the blood of *E. coli*-induced mastitis mice were significantly greater (*p* < 0.01), and the RBCs and HGB levels were also significantly greater (*p* < 0.05). Lymphocytes have specific immune recognition functions that are fundamental to immune processes, such as anti-infection and antitumor effects. An increase in the number of lymphocytes is related mainly to acute infection [31]. Monocytes, the largest white blood cells in the blood, are often involved in the defense and immune processes of the body. An increase in the number of monocytes suggests inflammation in the body. Red blood cells and hemoglobin are important media for transporting oxygen in the body. An increase in the number of RBC indicates a decrease in blood volume and blood concentration, whereas an increase in the number of HGB indicates hypoxia [32]. The results revealed that inflammation and hypoxia occurred in the mastitis model mice. After DBD treatment, the percentages of Lym, Mon%, the number of RBC, and HGB were normal in mastitis mice, which suggested that DBD can alleviate inflammation and hypoxia in these mice.

The most basic pathological changes associated with mastitis are inflammatory reactions and cell infiltration in breast tissue. We examined pathological sections of mammary tissue by staining and revealed that the model group had an incomplete mammary acinar structure, the mammary epithelial cells were depleted, vascular congestion occurred, and the blood vessels and the breast tissue were filled with a significant amount of inflammatory cells. In the DBD-treated group, the degree of pathological alterations was lower, and fewer inflammatory cells were present. In general, DBD can alleviate breast tissue damage caused by *E. coli* infection. This outcome is comparable to those reported by Zou et al. and Li et al. in the treatment of mastitis in mice with antimicrobial peptides and maslinic acid. Both drugs can alleviate pathologic changes in breast tissue [33,34]. Typical inflammatory cytokines include TNF-α, IL-1β, IL-6, and IL-8, and the levels of these cytokines can reflect the level of inflammation in the body [35]. Several studies have reported that the levels of TNF-α, IL-1β, IL-6, and IL-8 in dairy cows with mastitis increase [36,37]. The findings of this study revealed that *E. coli* infection of mouse mammary gland tissue increased the levels of various inflammatory factors in mice and induced an inflammatory response, whereas the expression of these inflammatory factors decreased after oral administration of DBD, indicating that DBD inhibited the production of inflammatory factors caused by *E. coli* infection at the protein and mRNA levels and played a significant anti-inflammatory role, thus protecting mammary gland tissue. This result was also reflected in the study by Yin et al., who used lentinan to treat LPS-induced mastitis in mice. After treatment with lentinan, pathological damage to mammary gland tissue in mice decreases, and the production of proinflammatory factors decreases [38]. The main toxic component of *E. coli* is lipopolysaccharides. When LPS binds to its specific receptor TLR4, the TIR domain of TLR4 binds to the key adaptor protein MyD88 to change the configuration of TLR4 and stimulate the activation of the IKK complex, thereby activating IκBα kinase and promoting IκBα phosphorylation. Phosphorylated IκBα is pantothenate and degraded so that NF-κB is separated and activated from the NF-κB/IκBα complex. Phosphorylated IκBα is pantothenate and degraded so that NF-κB is separated and activated from the NF-κB/IκBα complex. Activated NF-κB regulates inflammatory genes to cut inactive interleukins and tumor necrosis factor precursors into active inflammatory factors, causing proinflammatory effects [39]. To investigate whether or not the anti-inflammatory effects of DBD are achieved through the regulation of the TLR4/NF-κB signaling pathway, we assessed the expression of key factors in the pathway. The results showed that DBD can reduce the expression of the TLR4, MyD88, and IKKβ proteins and decrease the phosphorylation levels of the NF-κB p65 and IκBα proteins, thus inhibiting the activation of the TLR4/NF-κB signaling pathway and attenuating the inflammatory process in vivo.

Several studies have shown that the degree of OS is closely associated with the inflammatory response [40,41]. The immune response activated during mastitis in dairy cows reduces the available energy in the mammary gland, and the high energy demand during lactation increases lipid mobilization, further destroying the immune function of dairy cows and aggravating the production of reactive oxygen species and active nitrogen. The accumulation of ROS further activates the NF-κB signaling pathway and promotes the release of inflammatory factors. To resist OS, the body activates the transcription of the antioxidant gene Nrf2, promotes the release of detoxification enzymes and antioxidants, enhances the antioxidant capacity of cells, and protects cells from oxidative stress damage. The detection of T-AOC, GSH, SOD, MDA, and NO reflects the oxidative and antioxidant levels of the organism [42]. T-AOC refers to the total antioxidant content, which is composed of different antioxidant chemicals and antioxidant enzymes, and T-AOC is generally used to evaluate the antioxidant capacity of bioactive substances [43]. Glutathione scavenges free radicals, has antioxidative effects, performs integrated detoxification, and helps the body maintain normal immune system function [44,45]. SOD is a crucial antioxidant and antiaging protein in organisms. This enzyme scavenges free radicals and has antioxidant, antiaging, and immunomodulatory effects [46]. The MDA content and NO level can reflect the peroxidation level of an organism. The final product of lipid oxidation is MDA [47], and the MDA content in the body can indicate the speed and severity of lipid peroxidation. NO is an important molecule in the body that also has cytotoxic effects. When OS occurs in tissue, NO reacts with peroxides to produce cytotoxic peroxynitrite (ONOO^–^) [48]. While treating LPS-induced mastitis in mice with caffeic acid, Yu et al. confirmed that caffeic acid can improve the redox state and inhibit the release of proinflammatory factors in the body [11]. During inflammation, high levels of NO react with superoxide anions to form ONOO^–^, resulting in the peroxidation of long-chain fatty acids in the cell membrane and the production of MDA [49]. An increase in lipid peroxidation has also been reported in bovine [50], sheep [51], and streptococcal-induced mastitis in mice. Muralidhar reported an increase in MDA levels and SOD and catalase (CAT) activity in mice with mastitis induced by *Staphylococcus aureus* [52], whereas nanosilver treatment significantly reduced the bacterial load and SOD and CAT activity in breast tissue [53]. High levels of COX-2 can be detected in the inflammatory response. When tissue or cell damage elicits an inflammatory response, the expression of COX-2 in inflammatory cells can be tens or even hundreds of times greater than that in healthy cells; thus, COX-2 is a key player in the inflammatory response. PPARγ plays an important role in regulating lipid metabolism, the inflammatory response, and cell differentiation; therefore, PPARγ activators are commonly used to treat metabolic diseases. Thus, by comparing the changes in T-AOC, GSH, SOD, MDA, NO, COX-2, and PPARγ in the breast tissue of the DBD treatment group and the model group, the effects of DBD on the antioxidant capacity and OS level of the body can be determined. In this study, after DBD was orally administered to mastitis model mice, the T-AOC increased significantly and the GSH content and SOD activity in the tissues increased. However, the MDA content in the breast tissues decreased, and the NO level in the serum decreased, with reduced COX-2 and elevated PPARγ gene expression. These findings indicate that DBD can improve the antioxidant defense of the body and alleviate the peroxidation caused by *E. coli* infection in mice. Nrf2/HO-1 is an endogenous antioxidant signaling pathway, the activation of which can resist OS damage caused by external stimulation. Our results revealed that DBD treatment increased the expression of Nrf2, HO-1, and NQO1 mRNAs and proteins in mouse breast tissue, activated the antioxidant response in vivo, and resisted oxidative damage. These results were similar to the effects of hydroxytyrosol on LPS-induced inflammation and OS in bovine mammary epithelial cells [54].

DBD has been used in China for more than 800 years. Many studies have shown that the main chemical components of DBD include polysaccharides, flavonoids, saponins, and organic acids. Polysaccharides can participate in immune regulation and signal transduction. Flavonoids have anti-inflammatory, antioxidant, and liver protective effects. The main functions of saponins include immunomodulatory, anti-inflammatory, and antioxidant effects; organic acids are involved in energy metabolism, biosynthesis, and antioxidant and antibacterial effects. Therefore, we believe that DBD has anti-inflammatory, anti-injury, and antioxidative effects through the action of various active ingredients, such as organic acids, saponins, flavonoids, and polysaccharides.

Because clinical trials involving large animals face the challenges of high research costs, long cycles, difficulty, uncontrollable factors, and high ethical and moral requirements, researchers usually use animal models to study the pathogenesis of diseases. Therefore, we chose to evaluate the effect and potential risk of new BM therapy by establishing a mouse mastitis model, which can reduce research costs, improve research efficiency, and accelerate research progress. The method of using mice to construct models has certain limitations. These include physiological and metabolic differences between species, genetic and genetic background problems, pathological differences, and complexity. Researchers need to fully understand these limitations and combine other research methods to improve the reliability and extrapolation of the research and ensure its scientific validity and feasibility.

## 5. Conclusions

The findings of this study revealed a significant increase in inflammatory and OS responses in mice with *E. coli* infection-induced mastitis. Additionally, DBD plays a strong role in the treatment of mastitis in mice. In addition to alleviating the inflammatory response, it can improve the antioxidant capacity of the body to treat mastitis. Additionally, DBD exerts its anti-inflammatory and antioxidant effects by inhibiting the TLR4/NF-κB signaling pathway and activating the antioxidant Nrf2/HO-1 signaling pathway. These results provide important information for veterinarians regarding the use of DBD in the clinical management of mastitis in dairy cows.

## Figures and Tables

**Table 1:** List of primers.

Gene	Primers Sequences (5′→3′)	Gene Accession Number
TNF-α	F:ACGGCATGGATCTCAAAGAC R:GTGGGTGAGGAGCACGTAGT	NC_000083.7
IL-1β	F:GCCACCTTTTGACAGTGATGAG R:ATGTGCTGCTGCGAGATTTG	NC_000068.7
IL-6	F:CCCCAATTTCCAATGCTCTCC R:CGCACTAGGTTTGCCGAGTA	NC_000071.7
IL-8	F:GTCCTTCCTACCCCAATTTCCA R:TAACGCACTAGGTTTGCCGA	NC_000075.7
TLR4	F:GATCTGAGCTTCAACCCCTTG R:TGCCATGCCTTGTCTTCAAT	NC_000070.7
NF-κB p65	F:CGAGTCTCCATGCAGCTACG R:TTTCGGGTAGGCACAGCAATA	NC_000085.7
MyD88	F:CCCACTCGCAGTTTGTTG R:TGCCTCCCAGTTCCTTTG	NC_034599.1
IKKβ	F:TCAAGCAATGCCGACAGGAG R:CGATGTCACTCAGCAGGGTAAG	NC_000074.7
COX-2	F:ATAGACGAAATCAACAACCCCG R:GGATTGGAAGTTCTATTGGCAG	NC_000067.7
PPARγ	F:AAGAAGCGGTGAACCACTGA R:GGAATGCGAGTGGTCTTCCA	NC_000072.7
Nrf2	F:CAGCATGTTACGTGATGAGG R:GCTCAGAAAAGGCTCCATCC	NC_000068.8
HO-1	F:ACATTGAGCTGTTTGAGGAG R:TACATGGCATAAATTCCCACTG	NC_000074.6
NQO1	F:TGAGCCCGGATATTGTAGCTGA R:GCATACGTGTAGGCGAATCCTG	NC_058382.1
GAPDH	F:AGGTCGGTGTGAACGGATTTG R:TGTAGACCATGTAGTTGAGGTCA	NC_034591.1

## Data Availability

The data presented in this study are available on request from the corresponding author.

## Supplementary Materials

The following are available online at https://www.mdpi.com/article/10.3390/vetsci12030227/s1, S1: Western blots raw data showing all bands with molecular weight markers for corre-sponding figures.

## Abbreviations

The following abbreviations are used in this manuscript:

BM	Bovine mastitis
DBD	Danggui buxue decoction
*E. coli*	*Escherichia coli*
OS	Oxidative stress
