# Supplementary figures and images for: Danggui Buxue Decoction Alleviates Inflammation and Oxidative Stress in Mice with Escherichia coli-Induced Mastitis
Source: Vet Sci. 2025 Mar 2;12(3):227. doi: 10.3390/vetsci12030227 (PMC11946834; doi:10.3390/vetsci12030227)

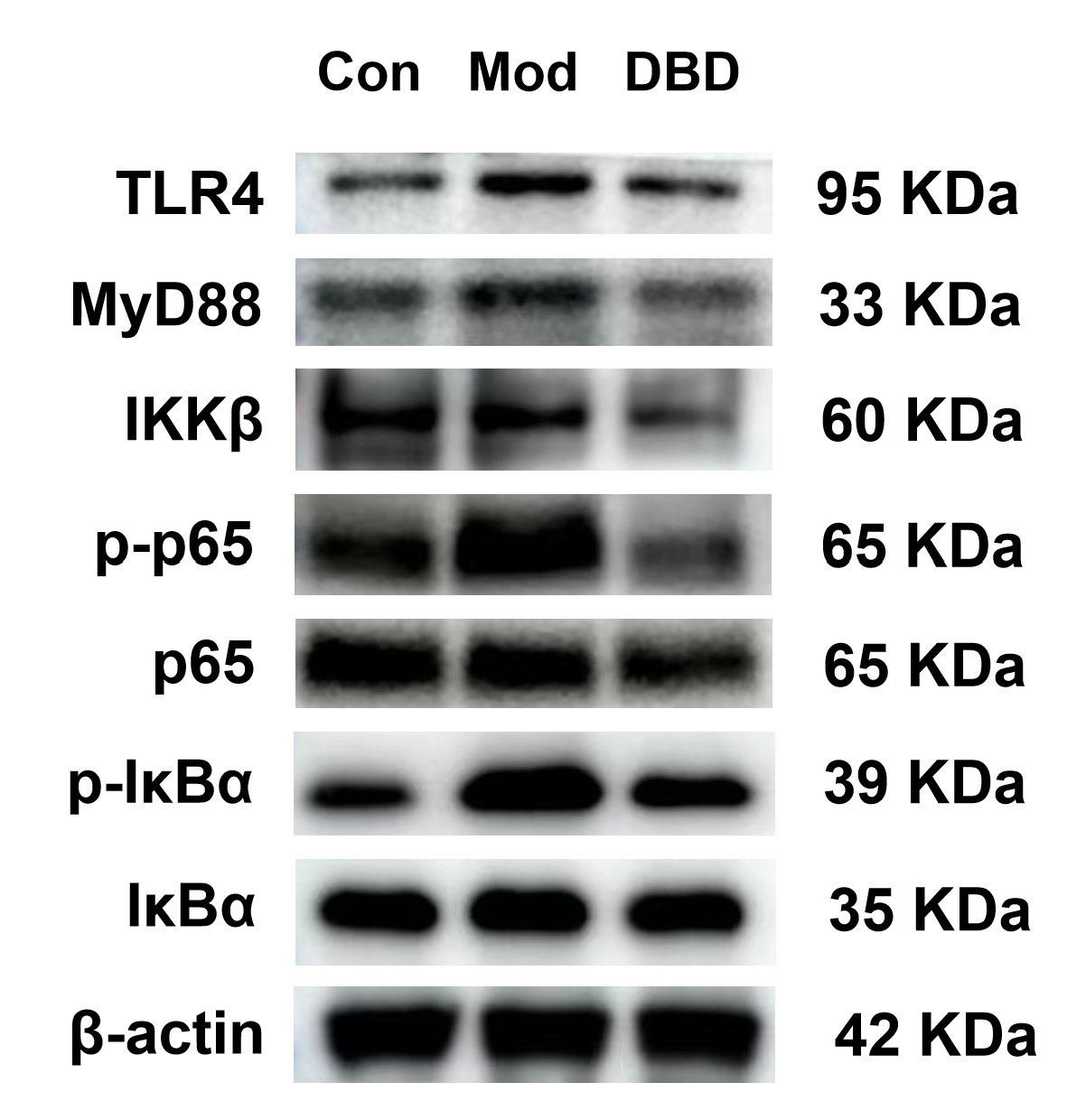

Supplement: Supplementary file 1 [file vetsci-12-00227-s001.zip › S1-WB Images/F11(B).tiff]

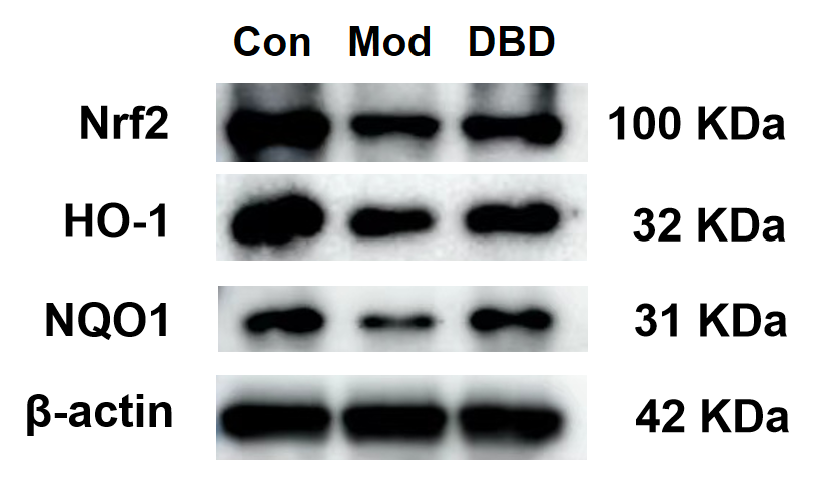

Supplement: Supplementary file 1 [file vetsci-12-00227-s001.zip › S1-WB Images/F13(B).tiff]

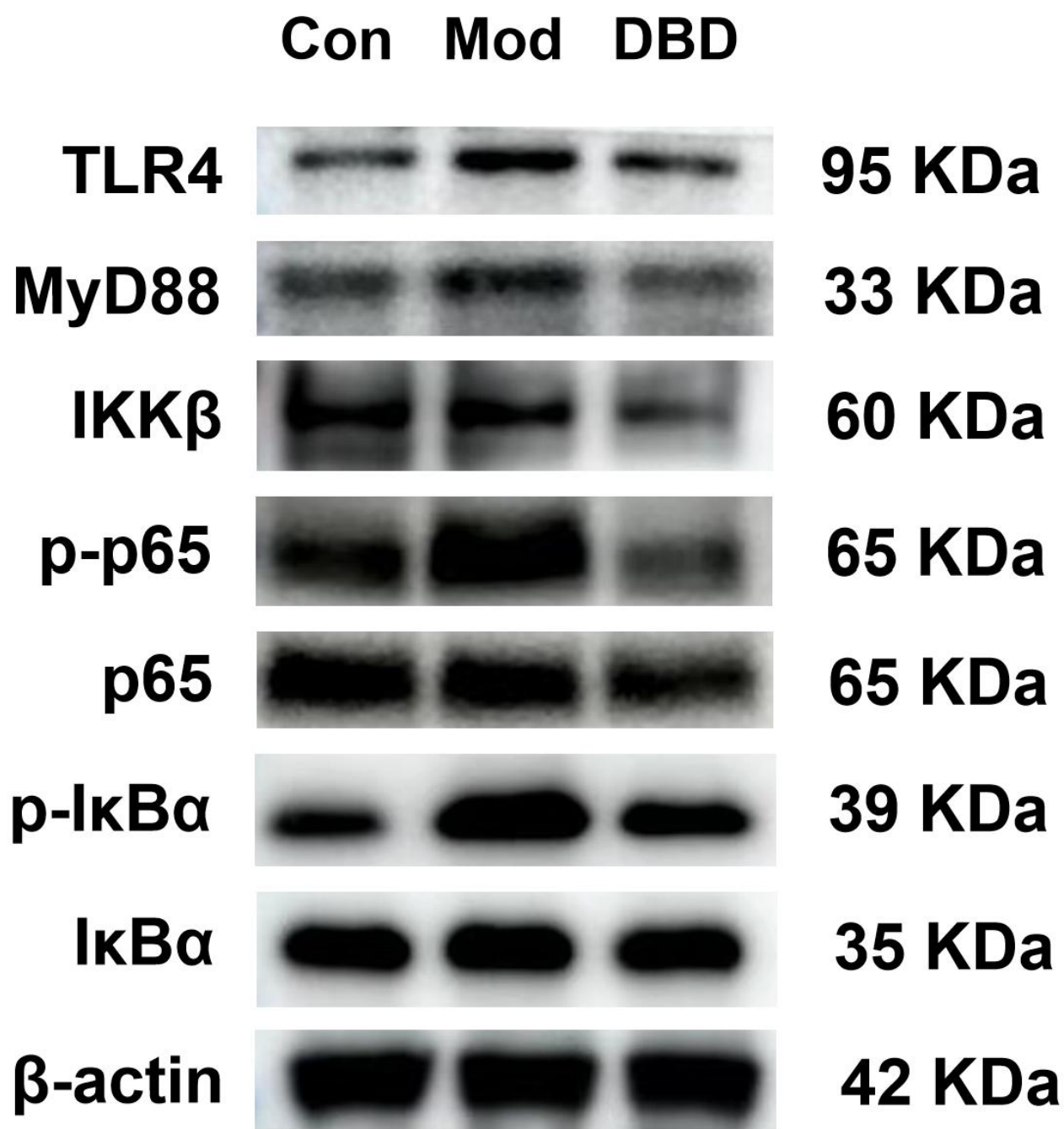

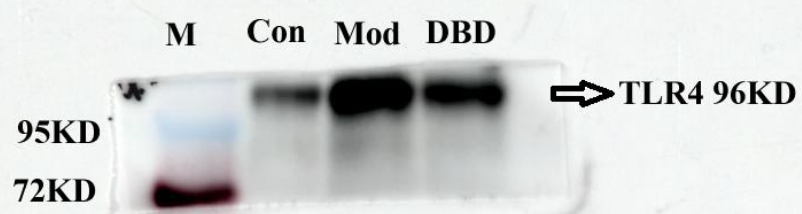

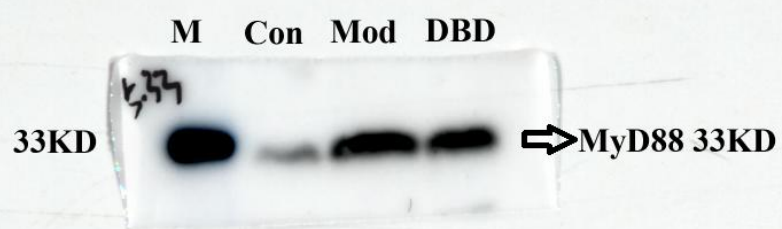

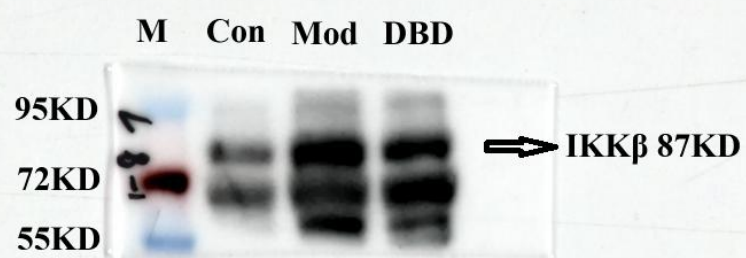

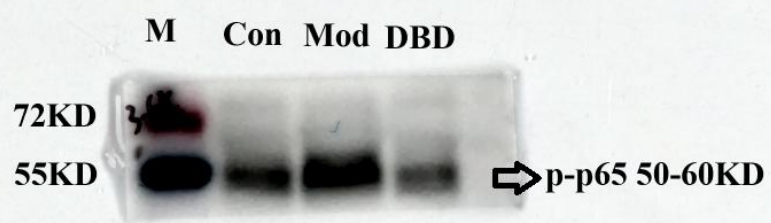

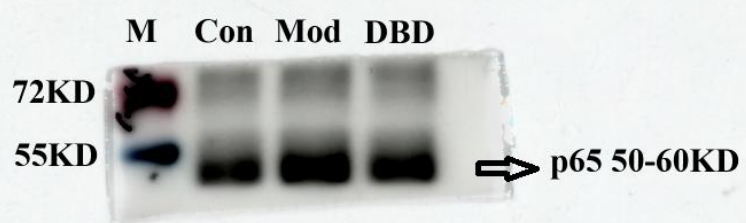

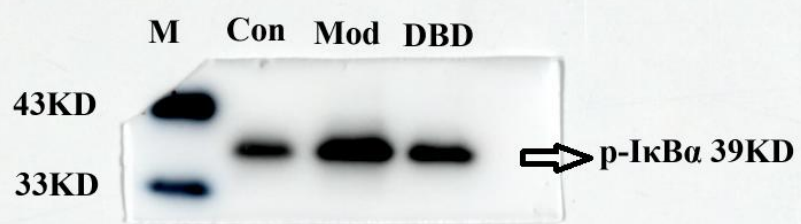

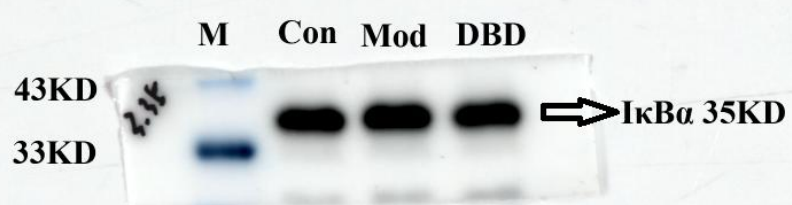

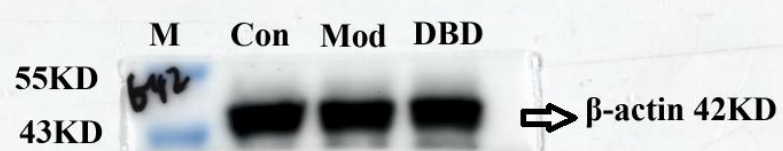

Supplement: Supplementary file 1 [file vetsci-12-00227-s001.zip › S1-WB Images/Fig11(B).pdf]

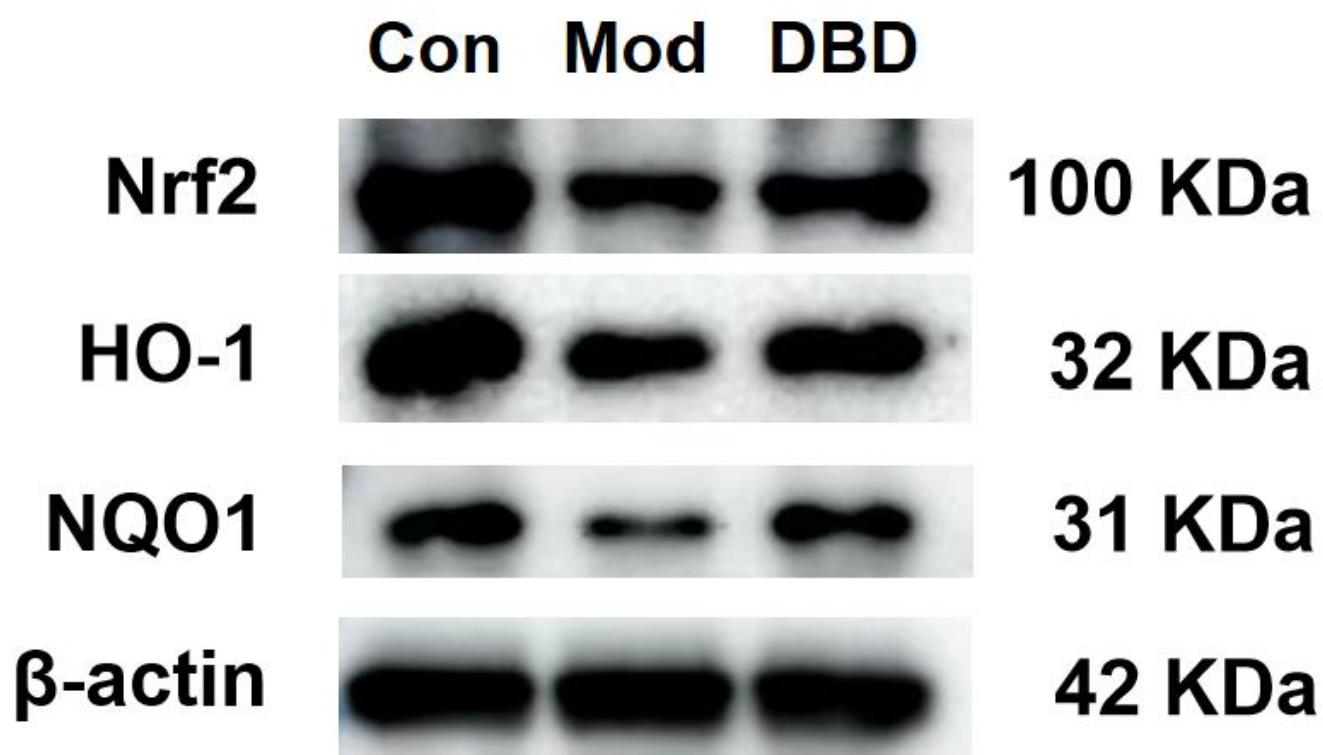

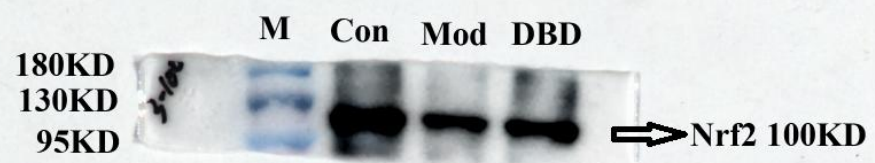

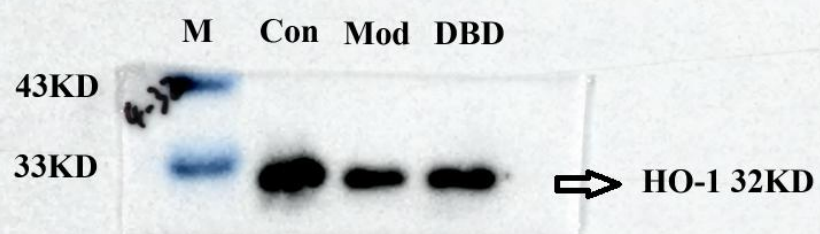

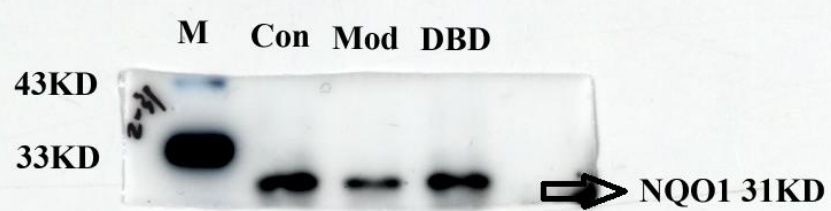

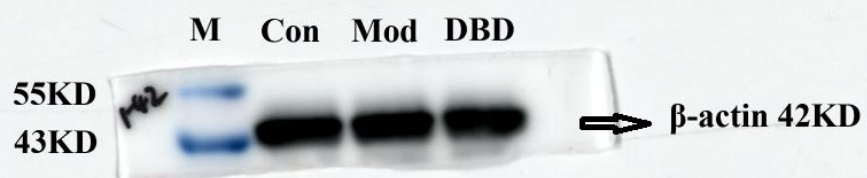

Supplement: Supplementary file 1 [file vetsci-12-00227-s001.zip › S1-WB Images/Fig13(B).pdf]

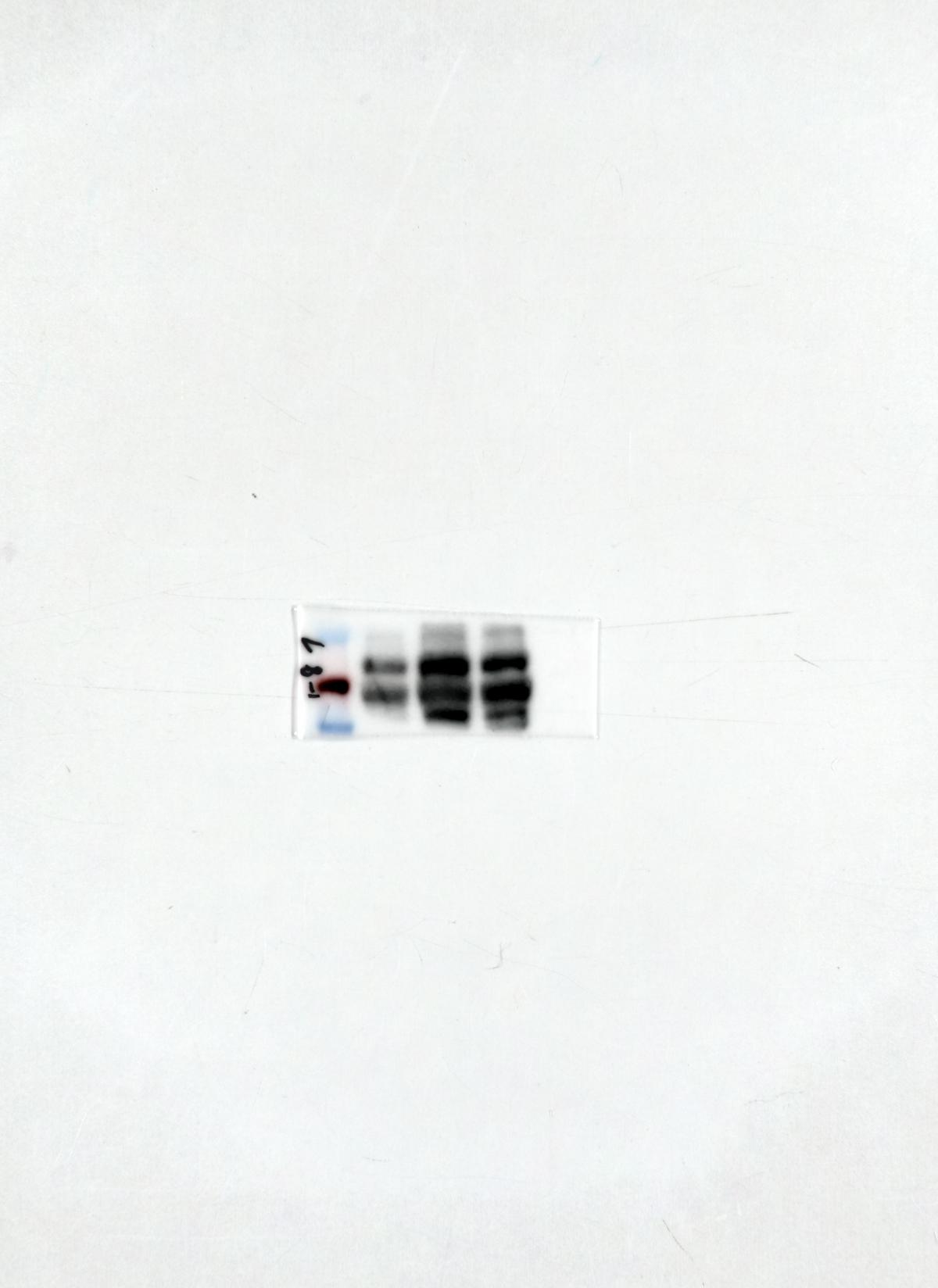

Supplement: Supplementary file 1 [file vetsci-12-00227-s001.zip › S1-WB Images/Figure 11-IKKa┬-60KD.tiff]

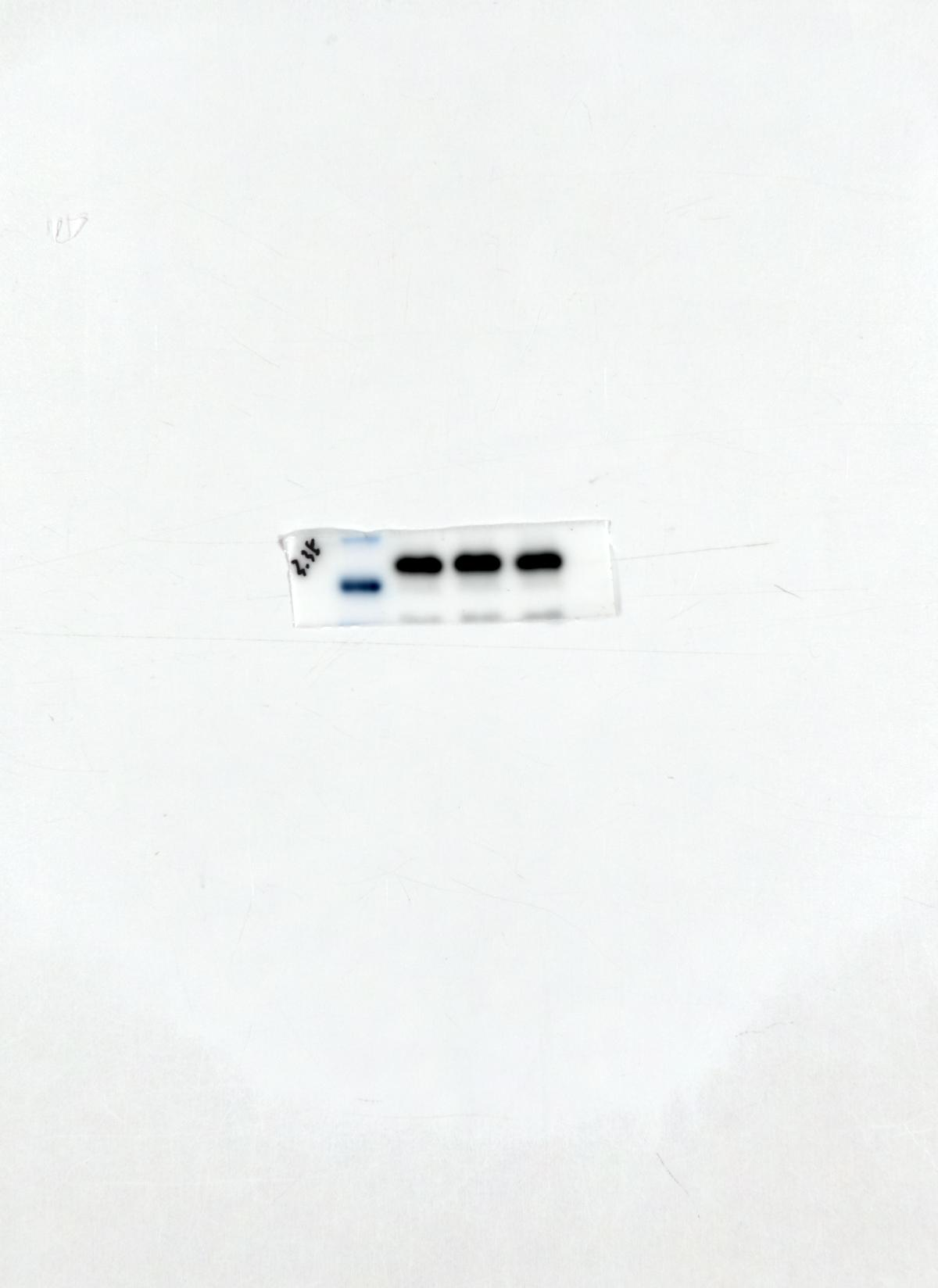

Supplement: Supplementary file 1 [file vetsci-12-00227-s001.zip › S1-WB Images/Figure 11-Ia╩Ba┴-35KD.tiff]

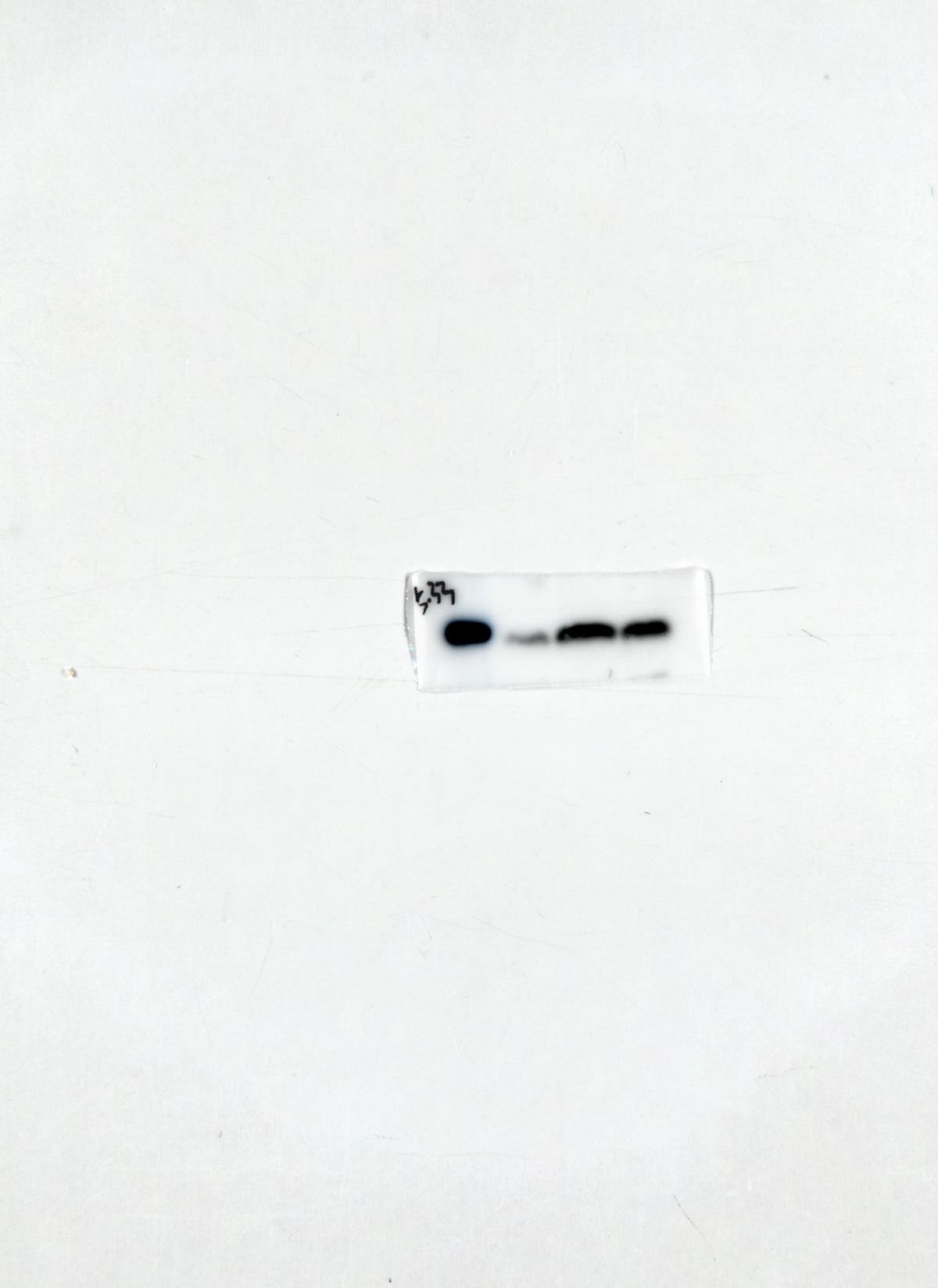

Supplement: Supplementary file 1 [file vetsci-12-00227-s001.zip › S1-WB Images/Figure 11-MyD88-33KD.tiff]

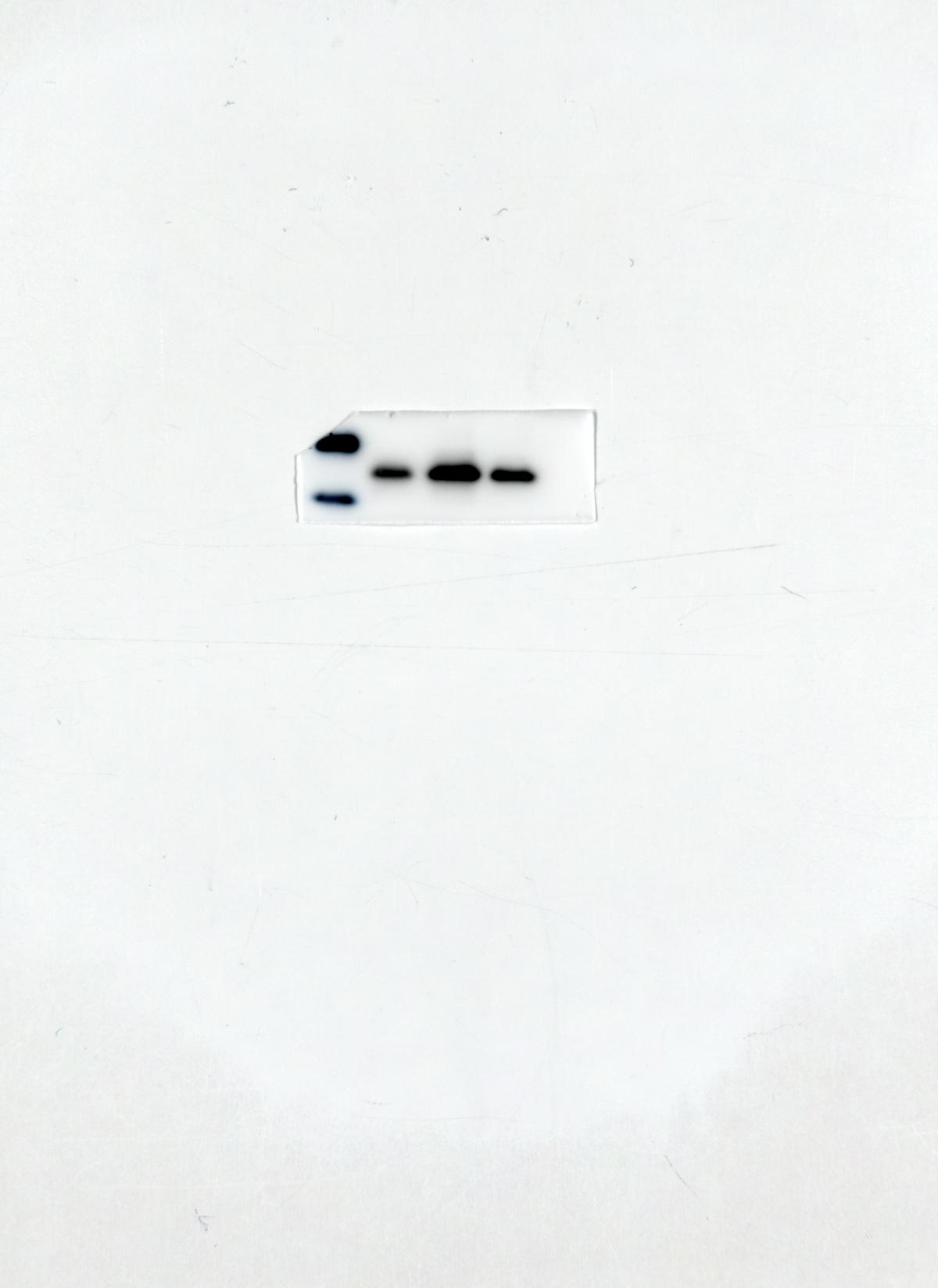

Supplement: Supplementary file 1 [file vetsci-12-00227-s001.zip › S1-WB Images/Figure 11-p-Ia╩Ba┴-39KD.tiff]

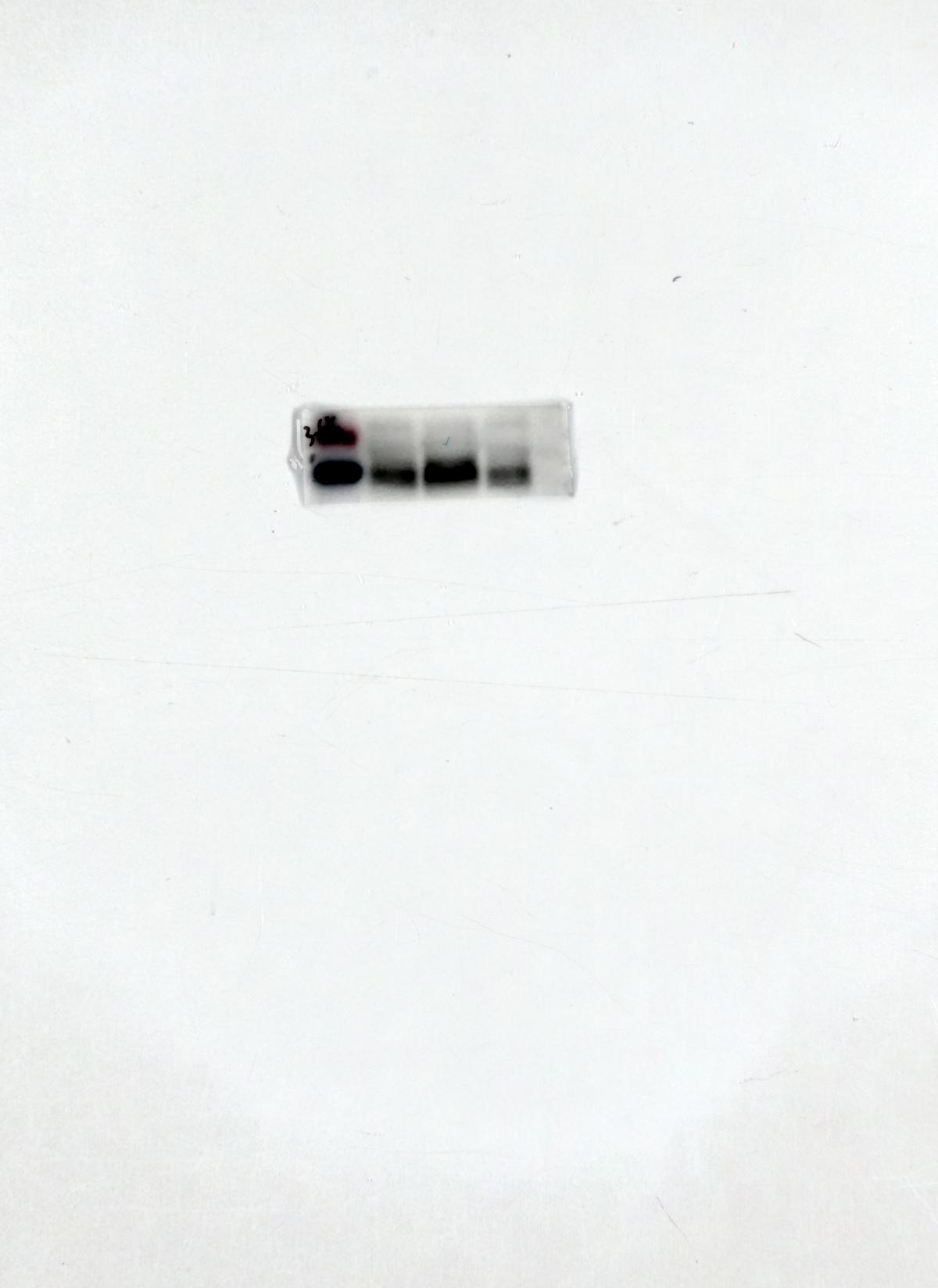

Supplement: Supplementary file 1 [file vetsci-12-00227-s001.zip › S1-WB Images/Figure 11-p-p65-65KD.tiff]

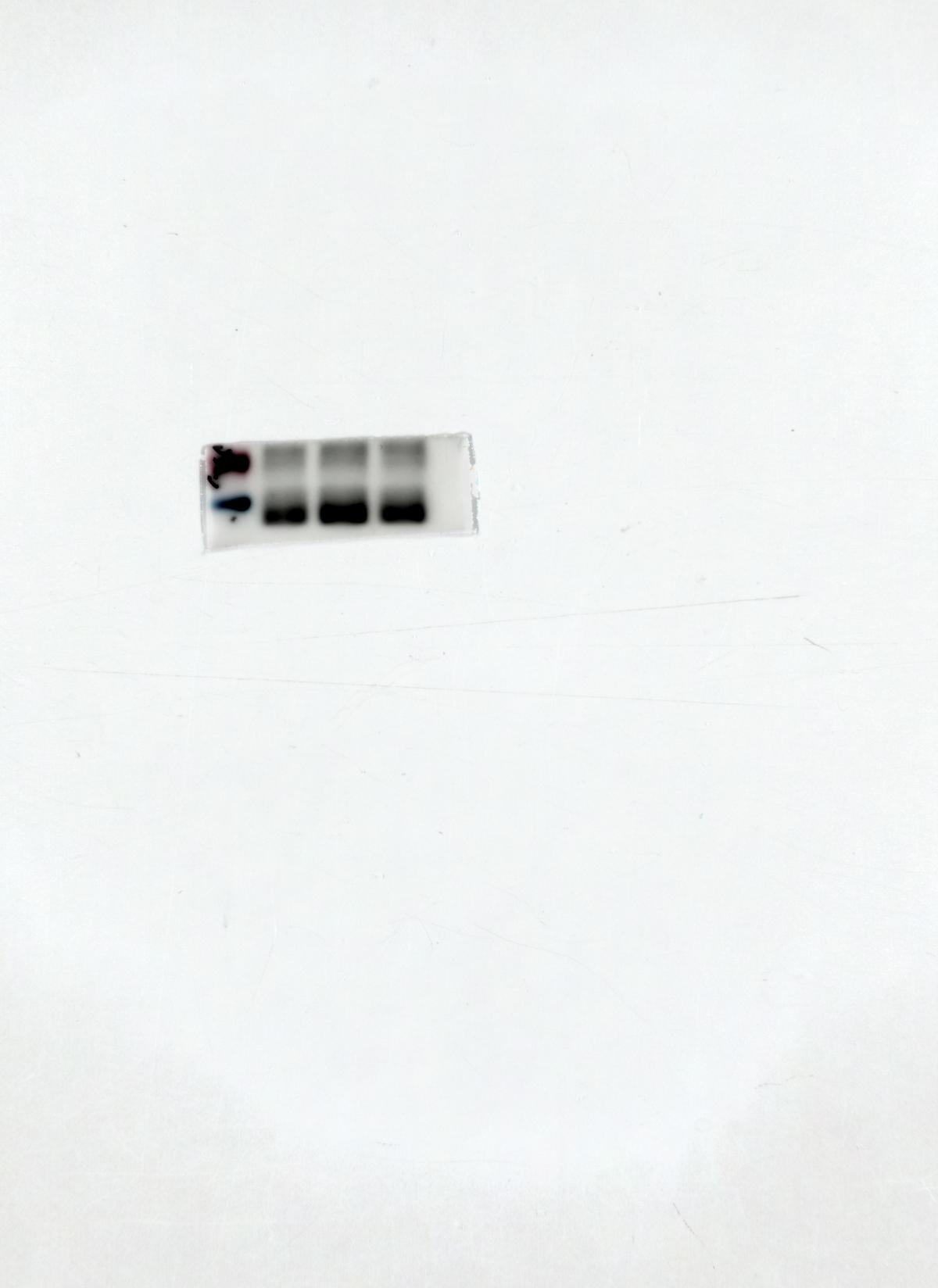

Supplement: Supplementary file 1 [file vetsci-12-00227-s001.zip › S1-WB Images/Figure 11-p65-65KD.tiff]

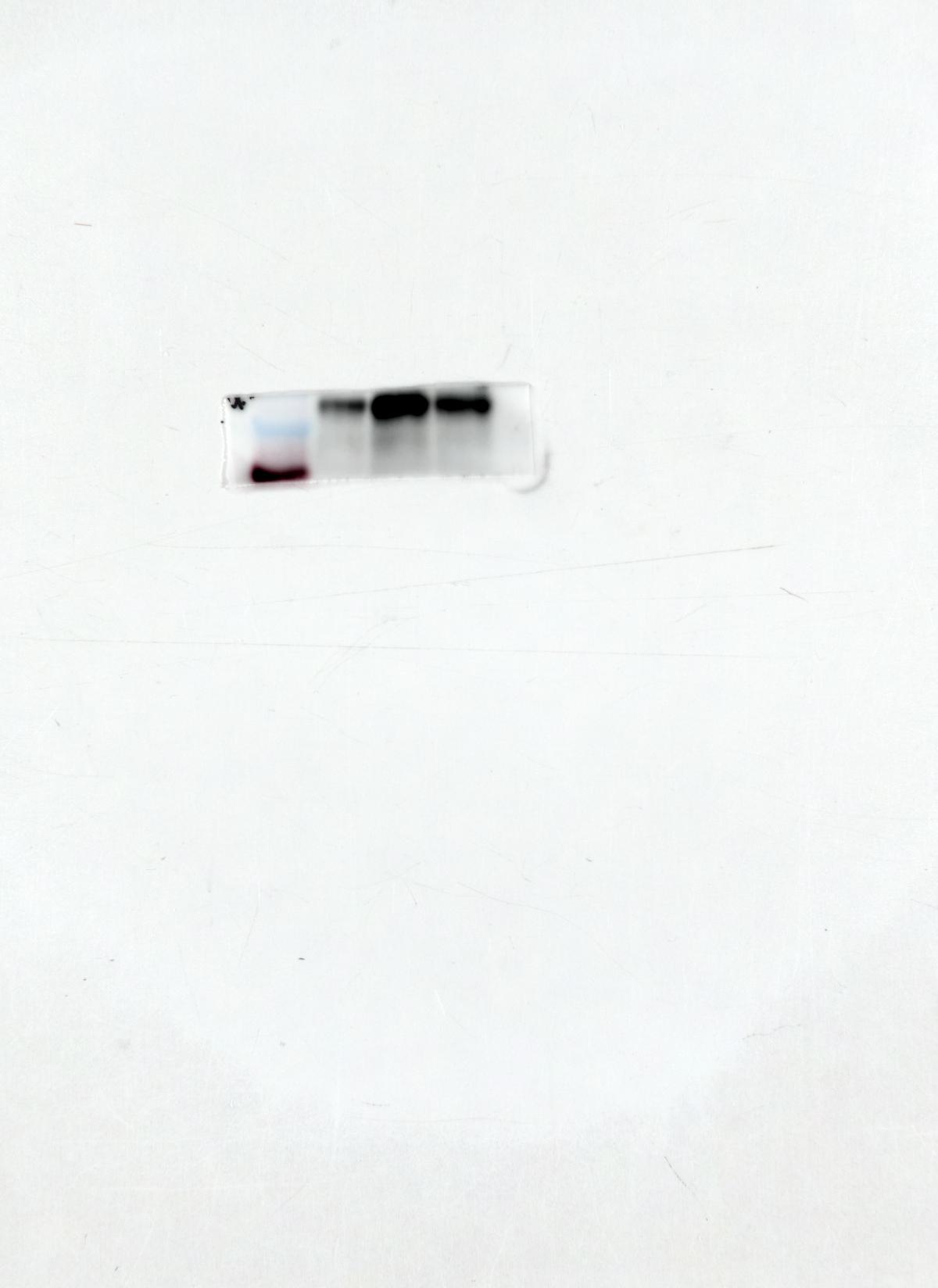

Supplement: Supplementary file 1 [file vetsci-12-00227-s001.zip › S1-WB Images/Figure 11-TLR4-96KD.tiff]

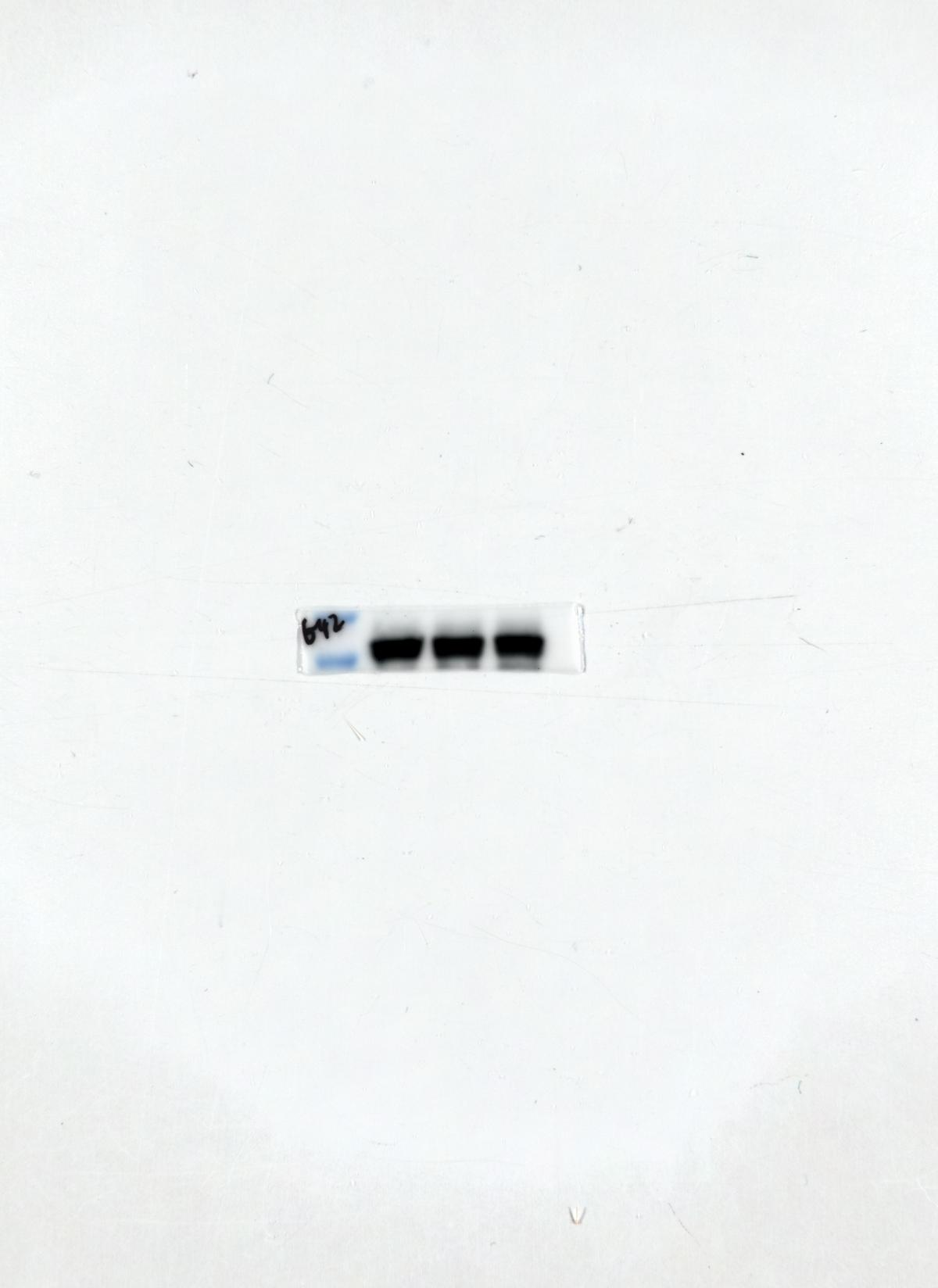

Supplement: Supplementary file 1 [file vetsci-12-00227-s001.zip › S1-WB Images/Figure 11-a┬-actin-42KD.tiff]

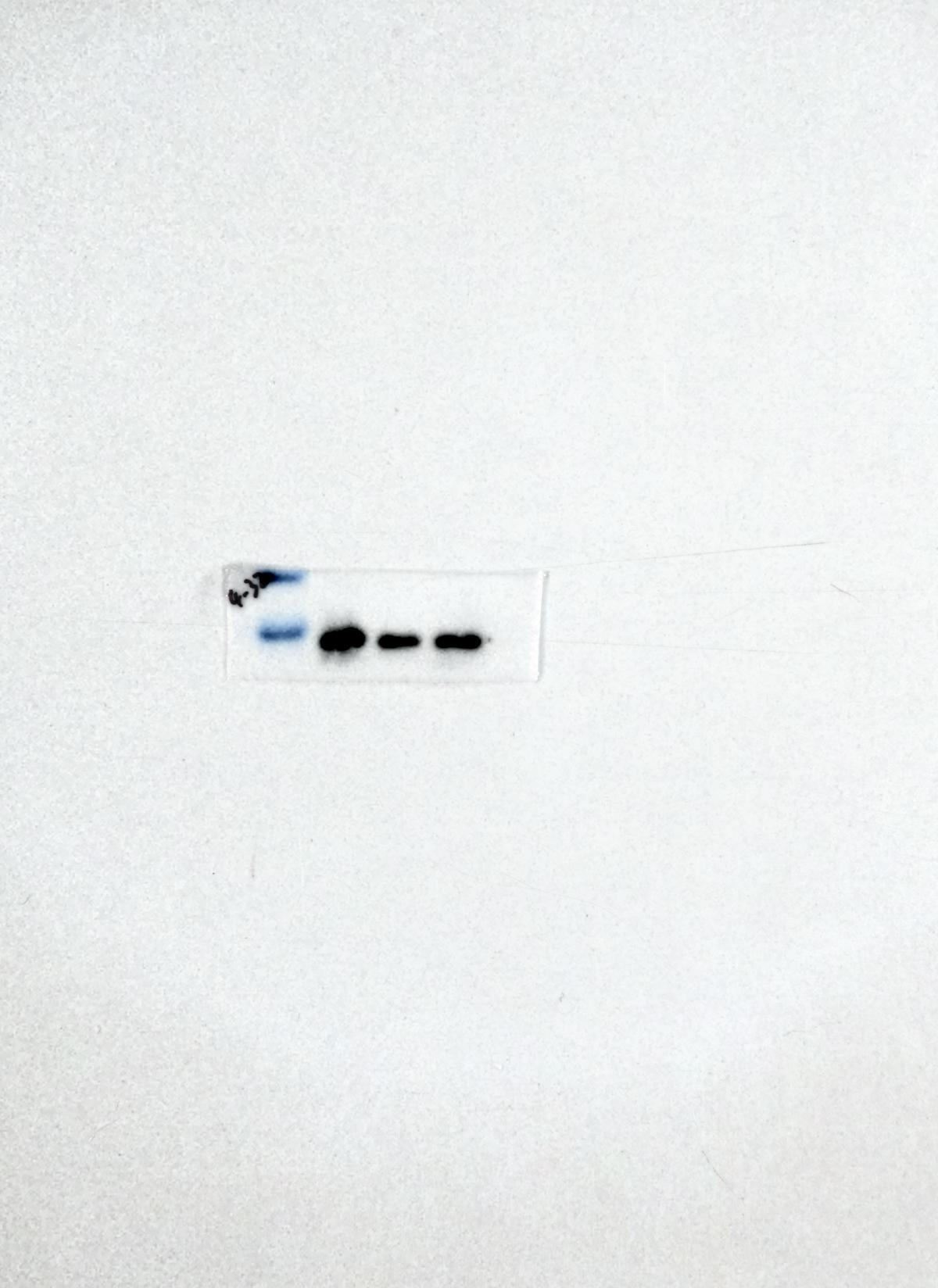

Supplement: Supplementary file 1 [file vetsci-12-00227-s001.zip › S1-WB Images/Figure 13-HO-1-32KD.tiff]

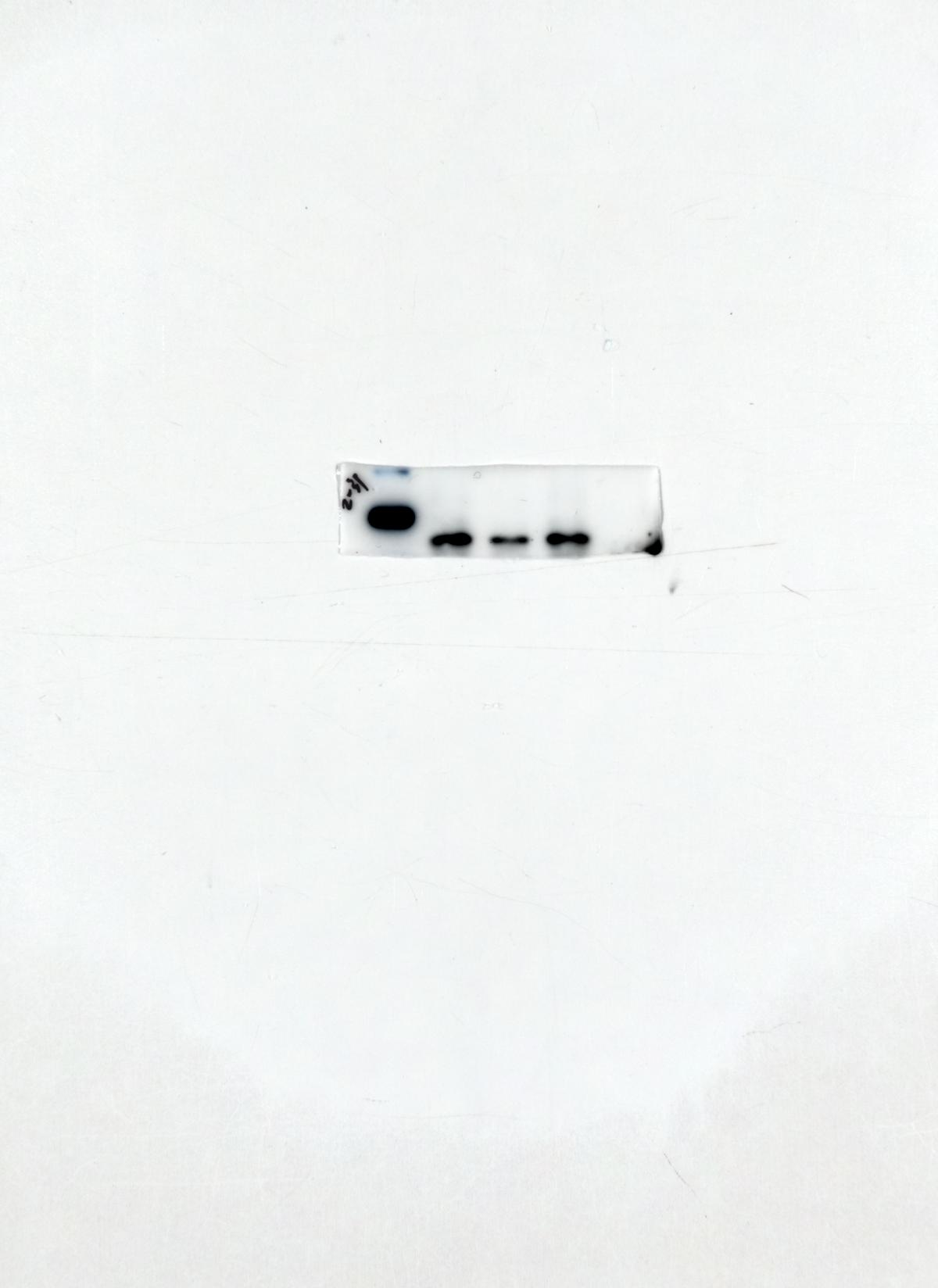

Supplement: Supplementary file 1 [file vetsci-12-00227-s001.zip › S1-WB Images/Figure 13-NQO1-31KD.tiff]

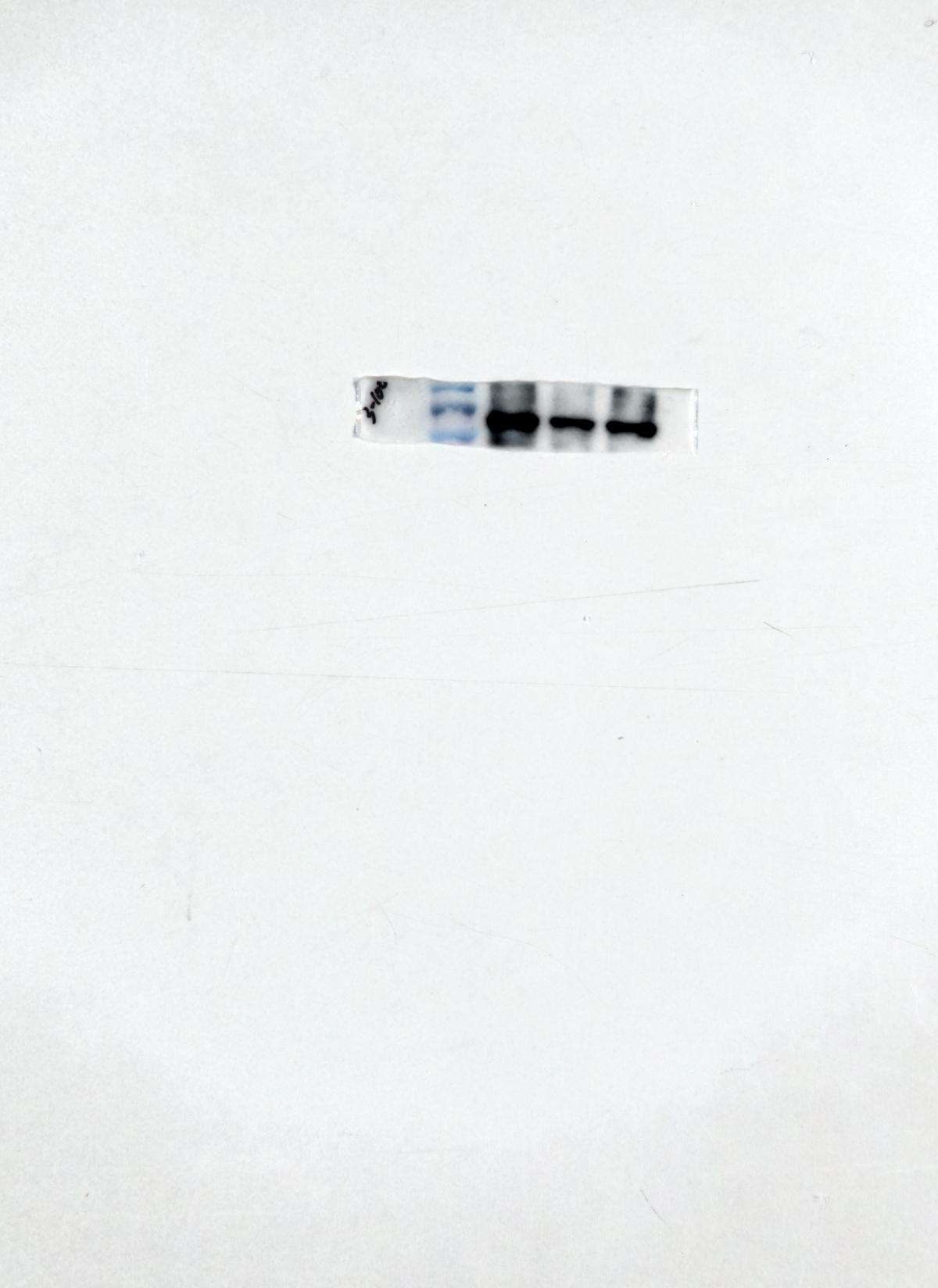

Supplement: Supplementary file 1 [file vetsci-12-00227-s001.zip › S1-WB Images/Figure 13-Nrf2-100KD.tiff]

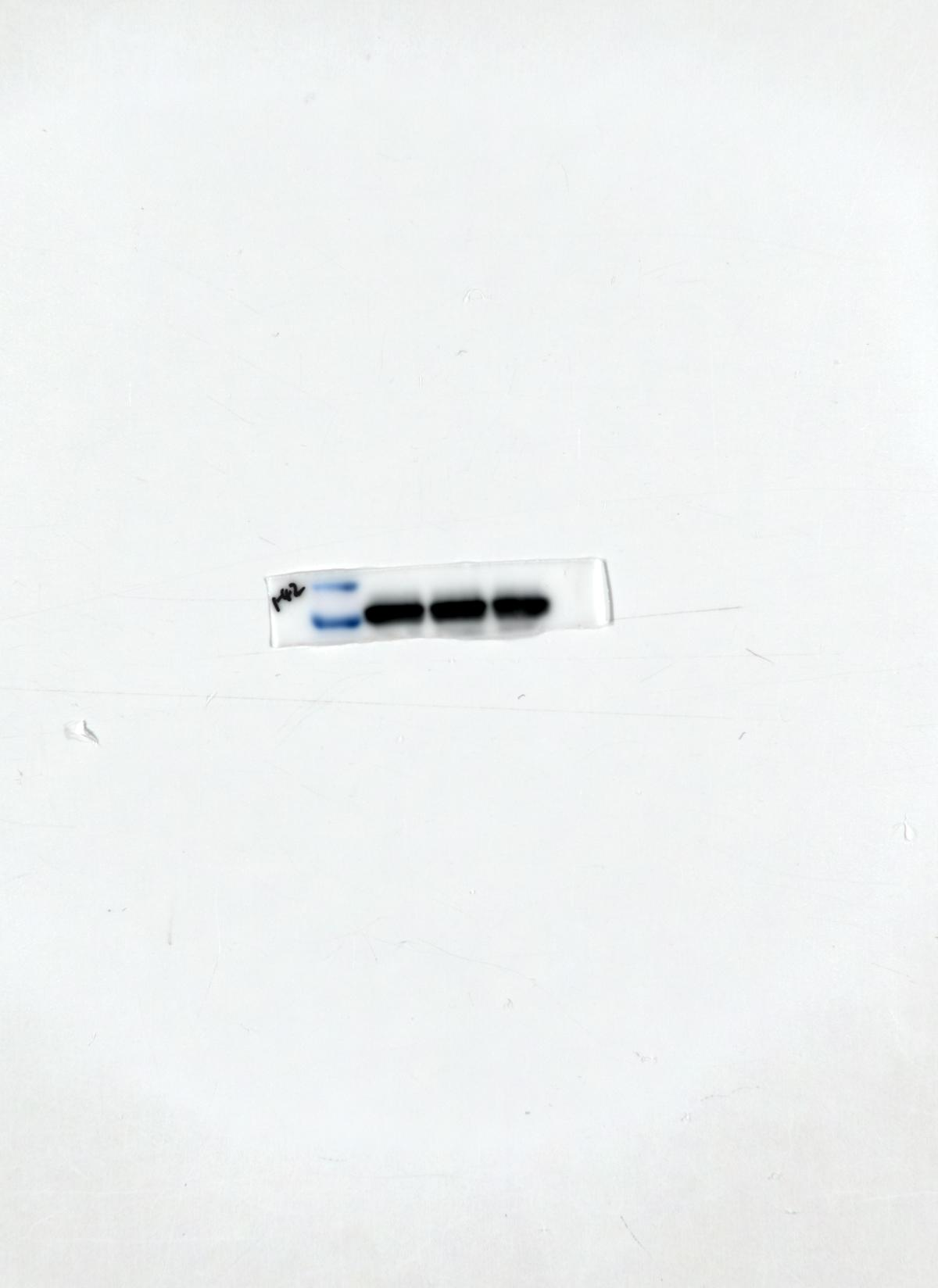

Supplement: Supplementary file 1 [file vetsci-12-00227-s001.zip › S1-WB Images/Figure 13-a┬-actin-42KD.tiff]
